# Adaptation to acetaminophen exposure elicits major changes in expression and distribution of the hepatic proteome

**DOI:** 10.1038/srep16423

**Published:** 2015-11-26

**Authors:** R. Eakins, J. Walsh, L. Randle, R. E. Jenkins, I. Schuppe-Koistinen, C. Rowe, P. Starkey Lewis, O. Vasieva, N. Prats, N. Brillant, M. Auli, M. Bayliss, S. Webb, J. A. Rees, N. R. Kitteringham, C. E. Goldring, B. K. Park

**Affiliations:** 1 MRC Centre for Drug Safety Science, University of Liverpool, Liverpool L69 3GE, UK; 2 Liverpool John Moores University, Byrom Street, Liverpool, L3 3AF, UK; 3 Almirall S. A. R&D Centre, Barcelona, Spain; 4 Institute of Integrative Biology, University of Liverpool, Liverpool, L69 7ZL, UK; 5 AstraZeneca R&D, Innovative Medicines, Personalised Healthcare & Biomarkers, Translational Science Centre, Science for Life Laboratory, Solna, Sweden

## Abstract

Acetaminophen overdose is the leading cause of acute liver failure. One dose of 10–15 g causes severe liver damage in humans, whereas repeated exposure to acetaminophen in humans and animal models results in autoprotection. Insight of this process is limited to select proteins implicated in acetaminophen toxicity and cellular defence. Here we investigate hepatic adaptation to acetaminophen toxicity from a whole proteome perspective, using quantitative mass spectrometry. In a rat model, we show the response to acetaminophen involves the expression of 30% of all proteins detected in the liver. Genetic ablation of a master regulator of cellular defence, NFE2L2, has little effect, suggesting redundancy in the regulation of adaptation. We show that adaptation to acetaminophen has a spatial component, involving a shift in regionalisation of CYP2E1, which may prevent toxicity thresholds being reached. These data reveal unexpected complexity and dynamic behaviour in the biological response to drug-induced liver injury.

Acetaminophen (paracetamol, APAP) overdose is the leading cause of acute liver failure in the USA and UK, resulting in over 600 deaths a year in these countries^1^,^2^. Whilst a single dose of 10–15 g is likely to result in severe liver damage^3^, prolonged exposure to acetaminophen has been shown to result in autoprotection in some patients, such that daily doses even in excess of 10 g have apparently little adverse effect. In one extreme example, APAP-induced autoprotection was demonstrated in an adult male addicted to the analgesic Percocet (APAP formulated with oxycodone), who consumed up to 65 g per day of APAP^4^. In addition, volunteers administered a daily therapeutic dose (4 g) of APAP displayed elevations in circulating liver enzymes (clinical markers of liver injury), which then resolved^5^. Autoprotection is therefore likely to be an important human defensive mechanism to prevent progressive injury resulting from drug toxicity.

Autoprotection to APAP has also been recapitulated in animal models: in mice, daily escalating doses can tolerize against liver damage within a week of treatment^4^. Our knowledge of the mechanism of hepatic adaptation is limited, and focus to date has been on select proteins implicated in APAP toxicity. APAP liver damage is caused by a metabolite – N-acetyl-p-benzoquinoneimine (NAPQI) – thus enzymes involved in the formation or detoxification of NAPQI are likely to be involved in the adaptive response. In particular, cytochrome P450 (CYP) 2E1 which activates APAP to NAPQI and the multidrug resistance-associated proteins ABCC3 and ABCC4 (also known as MRP3 and MRP4), which transport APAP and other xenobiotics out of cells, have been implicated in the autoprotection^4^,^6^. Alternatively, proteins involved in the cell’s natural defence systems, including those regulating glutathione (GSH), may underlie the adaptation. A recent microarray study also linked the expression of a number of novel genes to the development of tolerance to APAP^7^. Induction of flavin-containing monooxygenase-3 (FMO3), an enzyme identified in this study that has not previously been associated with APAP metabolism, was subsequently shown to be protective in an APAP autoprotection model^8^.

Here, using a rat model, we have investigated this process and show that in fact the expression of as many as 30% of all proteins detected in the liver is altered during adaptation to APAP, and see a dramatic shift in the localisation of CYP2E1. This indicates that the process of adaptation to APAP-induced liver injury is more extensive and dynamic than previously thought.

## Results

**Figure 1:**
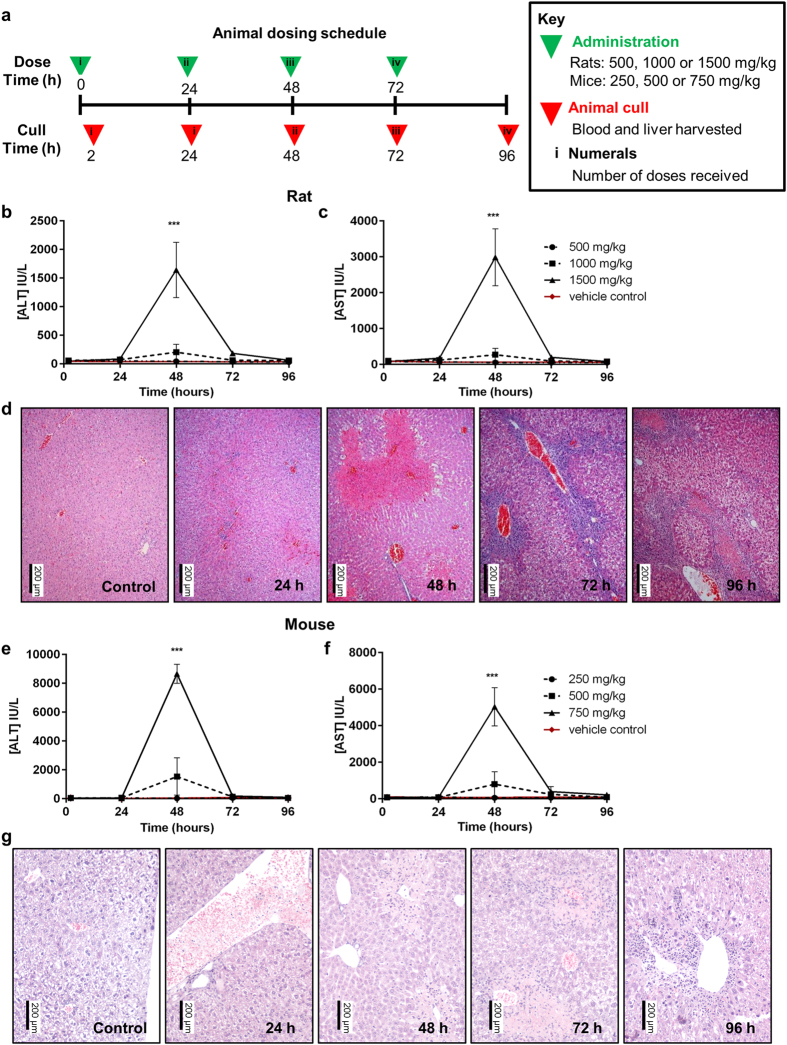
Autoprotection occurs in the rat and the mouse following repeat acetaminophen exposure. (**a**) Dosing protocol used in the study. (**b**) ALT and (**c**) AST were determined in rat serum (n = 6). Both biomarkers were significantly elevated in the 1500 mg/kg dose group alone (ANOVA with Tukey post-test, ***p < 0.001; 500 mg/kg circle, 1000 mg/kg square, 1500 mg/kg triangle, vehicle control diamond). (**d**) H&E staining of liver slices for groups of rats at each time-point in 1500 mg/kg group showed progression of injury (n = 4, representative images shown). (**e**) ALT and (**f**) AST were determined in mouse serum (n = 6). Both biomarkers were significantly elevated in the 750 mg/kg dose group alone (ANOVA with Tukey post-test, ***p < 0.001; 250 mg/kg circle, 500 mg/kg square, 750 mg/kg triangle, vehicle control diamond). (**g**) H&E staining of liver slices for groups of mice at each time-point in 750 mg/kg group showed progression of injury (n = 4, representative images shown).

We examined two separate species, rat and mouse, for adaptation to repeat APAP exposure, in order to ensure that this is not a species-selective process and therefore more likely to be relevant to man. The two models were selected because of the similar sensitivity of the rat to human APAP hepatotoxicity^9^,^10^,^11^, and because the mouse is more amenable to genetic modification in order to test the role of specific genes in the process. Rats were dosed orally with 500, 1000 or 1500 mg/kg APAP, and mice with 250, 500 or 750 mg/kg APAP. The doses were chosen in order to monitor autoprotection across a range of sub-toxic, threshold toxic and overtly toxic doses of APAP, to ensure that the drug exposure is relevant to what may occur in humans. Animals were either dosed once at 0 h with sacrifice at 2 h or 24 h, or at 24 h intervals for up to 72 h and sacrificed 24 h after the final dose administered. An outline of the dosing protocol is shown in Fig. 1a. At the 1500 mg/kg dose, at 48 h, rats exhibited rises in circulating liver enzymes, showing a peak serum alanine aminotransferase (ALT) rise 36-fold above vehicle controls, and a 33-fold serum aspartate aminotransferase (AST) rise over control (Fig. 1b,c). Both markers returned to normal levels by 96 h. Histopathology analyses were performed in order to validate the model of liver injury (representative images are shown, Fig. 1d). Although substantial hepatocellular damage was seen at 48 h, this injury had largely resolved by 72 h, despite the animals continuing to receive a daily toxic dose of APAP.

At the 750 mg/kg dose of APAP, at 48 h, mice also displayed a peak serum ALT rise, which reached 200-fold above vehicle controls, and a 67-fold serum AST rise over control (Fig. 1e,f). Both markers also returned to normal levels by 96 h. Although substantial hepatocellular damage was seen at 48 h (Fig. 1g), this injury had resolved considerably by 72 h, as in the rat model.

In order to reveal the breadth of change occurring in the liver during adaptation, we selected the rat model for comprehensive analysis using a global bioanalytical approach. While ALT levels were significantly elevated at 48 h in the rat (as they were in the mouse), indicative of substantial hepatocellular damage, the degree of overt liver tissue degeneration was low (in contrast to the mouse) as assessed by histopathology, thus allowing robust proteomic analysis. The technique of isobaric tags for relative and absolute quantification (iTRAQ) allows unambiguous identification and quantification of proteins expressed in a complex tissue matrix, and presents here a snapshot of the hepatoproteome at each of the time-points examined. Analysis of rat liver identified 2181 unique proteins, of which 1169 were common to all animals and all time-points, and were therefore amenable for statistical analysis and pathway mapping. Lists of significantly altered proteins are shown in Supplementary Tables 1a–d.

**Figure 2:**
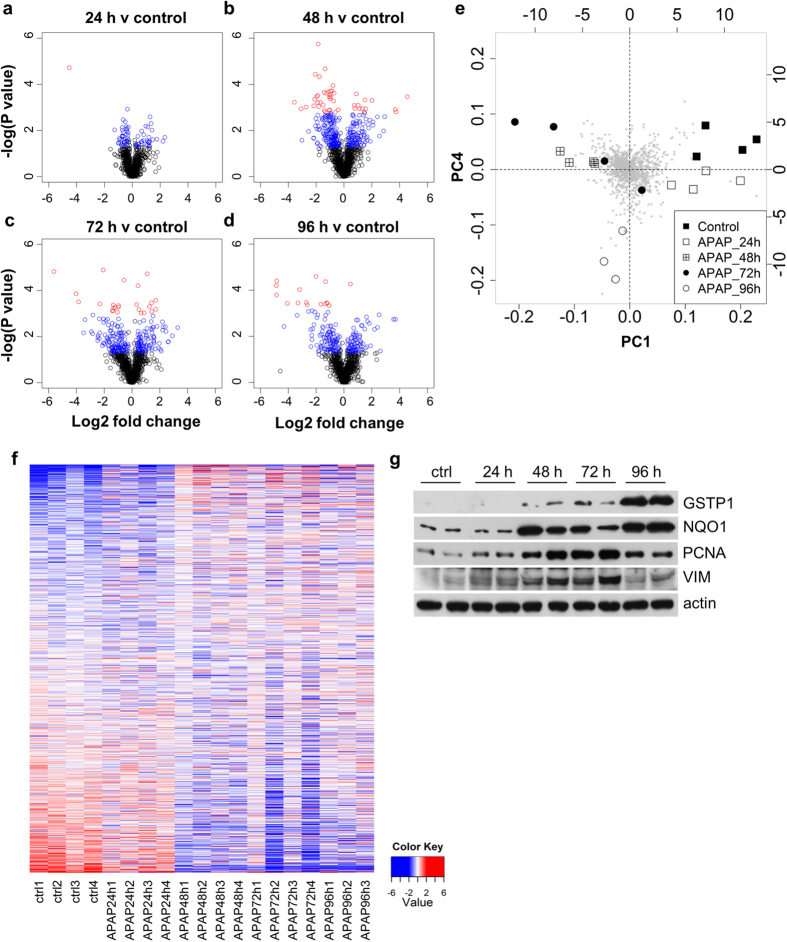
Widespread changes in protein abundance occur in rat liver following repeated acetaminophen exposure. (**a**–**d**) Volcano plots of all common proteins quantified by iTRAQ analysis, at each time-point (**a**) 24 h, (**b**) 48 h, (**c**) 72 h, (**d**) 96 h, relative to vehicle control. A complete list is provided in Supplementary Tables 1a–d. Coloured circles represent differential expression (blue - raw P value, p < 0.05; red – FDR, p ≤ 0.05). (**e**) Principal Components Analysis identified the greatest differences between single and repeat dose samples. (**f** ) Heat map representing the 1169 proteins common to all samples and all time-points identified distinct changes in protein abundance in repeat-dosed animals (red indicates increased abundance, blue indicates decreased abundance). (**g**) Western blots for GSTP1, NQO1, PCNA and VIM, performed in order to validate proteomic findings. Representative blots of two rats at each time-point are shown. Actin was used as a loading control.

Global changes at each time-point were visualised as volcano plots (Fig. 2a–d), in which significance (*y*) is plotted against fold change (*x*). Although changes can be seen at 24 h (Fig. 2a), at 48 h (Fig. 2b; peak toxicity) the volcano plots show the greatest change in protein abundance, as indicated by the number of blue points (raw p < 0.05) and red points (FDR ≤0.05). Large numbers of protein changes are still observed at 72 h (Fig. 2c) and 96 h (Fig. 2d). Principal Component (PC) analysis was performed to identify the proteins contributing to the clearest differences in the data set as a whole (Fig. 2e). Comparing PC1 to PC4 allowed separation into three distinct groups (in Fig. 2e, see control and 24 h to the top right, 48 h and 72 h to top left, and 96 h to the bottom of the plot), thereby identifying groups of proteins contributing to the major differences between these groups (Fig. 2e). These proteins are listed in Supplementary Table 2. Numerical descriptions of significant changes are shown in Table 1.

The subset of 1169 proteins common to all animals at every time-point is expressed as a heat map (Fig. 2f), which demonstrates the similarity in protein expression levels between control and single dose livers (24 h). By contrast, the profile of the same proteins in the repeat dose livers (48 h and 72 h) appears markedly changed. By 96 h a further shift in the protein expression profile is seen, consistent with the clustering shown in Fig. 2e. These observations are consistent with immunoblot data carried out on four proteins (Fig. 2g), selected from the mass spectrometric data for their different properties as sentinels of metabolic function or regeneration: Glutathione S-transferase P1 (GSTP1), NAD(P)H dehydrogenase [quinone] 1 (NQO1), Proliferating cell nuclear antigen (PCNA), and vimentin (VIM). NQO1 and GSTP1 are important enzymes in the detoxification of NAPQI, the toxic metabolite of APAP. PCNA is a marker of replication^12^, and indicates a surge in proliferative activity in the rats which peaks at 72 h. VIM is a classical marker of progenitor cells and is upregulated in cells that are undergoing epithelial to mesenchymal transition, a process implicated in wound healing and organ fibrosis^13^,^14^. Overall, the data indicate a much wider response in adaptation than has been posited previously, not only implicating proteins directly involved in APAP metabolism.

**Figure 3:**
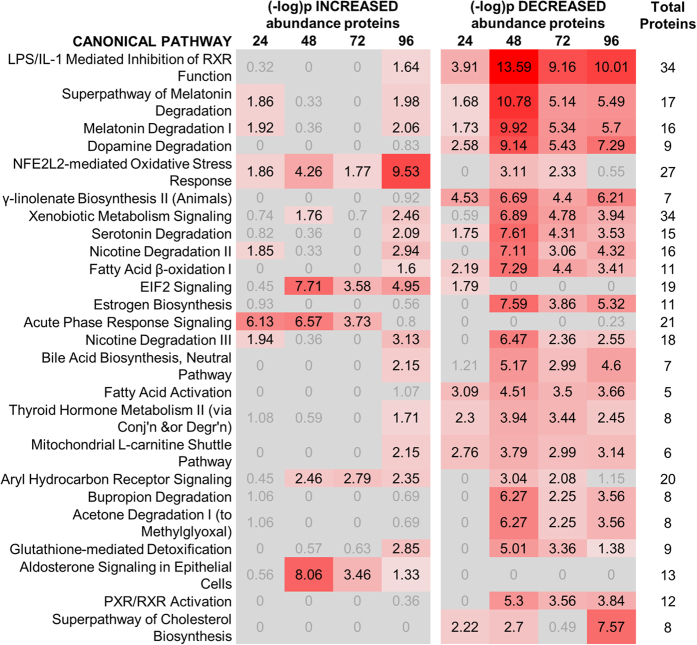
Analysis of the rat liver proteome reveals widespread pathway changes in response to repeat acetaminophen exposure. Ingenuity pathway analysis showing the top twenty-five most perturbed canonical pathways during the process of adaptation to APAP exposure. The left panel shows the significance of increased abundance proteins and the right panel show significance of reduced abundance proteins. The final column denotes the number of unique proteins identified per canonical pathway. Lists are shown in Supplementary Table 3. Values in red are significant with colour intensity proportional to significance.

Ingenuity pathway analysis identified the top twenty-five most perturbed canonical pathways during the process of adaptation to APAP (Fig. 3, a full list of proteins is shown in Supplementary Table 3). The changes highlight alterations in pathways that could be anticipated in our model, e.g. *NFE2L2-mediated oxidative stress response* and *the acute phase response* and others that were unexpected, e.g. *melatonin degradation and dopamine degradation*. This indicates, on a pathway-scale, that autoprotection to APAP involves a range of liver processes that are much more diverse than previously recognised.

**Figure 4:**
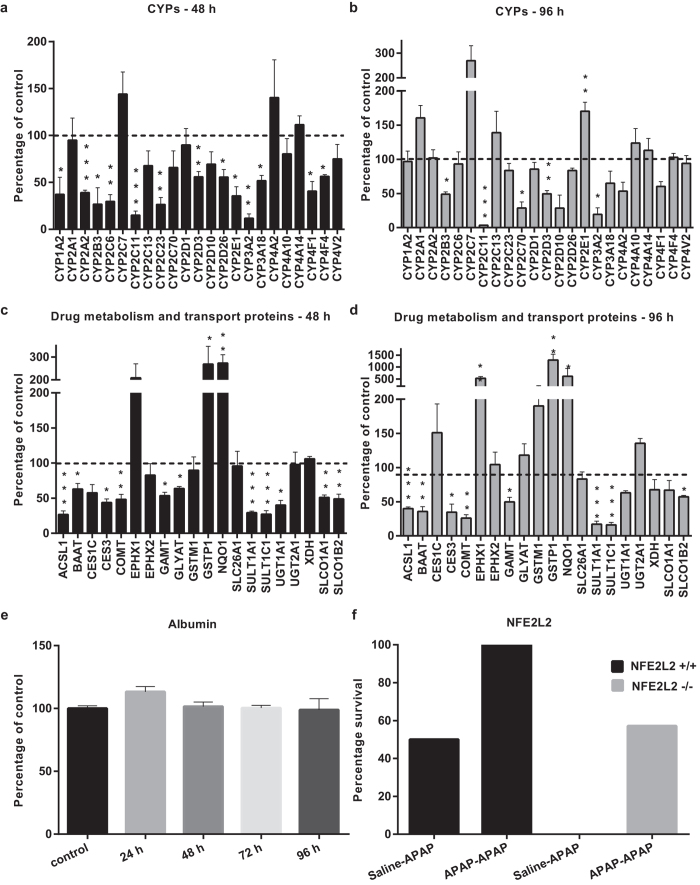
Investigation of key liver pathways identified by proteomic analysis in response to repeat acetaminophen exposure. In rats, while the abundance of phase I (CYP450) proteins at (**a**) 48 h and (**b**) 96 h, and phase II and III proteins at (**c**) 48 h and (**d**) 96 h was altered compared to control, serum albumin (**e**) remained statistically unchanged throughout the timecourse. Bars represent mean protein abundance (n = 3–4; percentage of control +SEM). Dashed lines indicate mean control value. Statistical analysis was performed using a linear model in the R programming environment (ANOVA with Dunnett’s post-test, *p < 0.05, **p < 0.01, ***p < 0.001). (**f**) Survival of NFE2L2^+/+^ and NFE2L2^−/−^ mice in APAP adaptation study. Bar chart comparing survival of toxic challenge after APAP or saline (0.9%) pretreatment in NFE2L2^+/+^ or NFE2L2^−/−^ animals. Mice were pretreated with incremental doses of APAP ranging from 150–600 mg/kg i.p. or vehicle control for 8 days followed by a final challenge of 1000 mg/kg APAP or vehicle on day 9.

Changes in the proteins responsible for the disposition of most drugs, the so-called Phase I, II and III proteins, were explored in more depth (Fig. 4a–d). Thirteen out of the twenty-three quantified CYP enzymes, the single most important set of proteins that govern how the liver initially processes a drug, were found to be present at lower abundance at 48 h. This included CYP2E1, which is largely expressed in the centrilobular region of the liver and CYP2C6, which is expressed across all zones of the liver (36% and 30% of control values, respectively), although both of these proteins increased after 96 h (Fig. 4a,b). The majority of detected phase I and II proteins decreased during the process of adaptation to APAP. Notable exceptions to this were NQO1 and GSTP1. Both of these enzymes were more abundant at 48 h (273% and 269% of control values respectively, Fig. 4c), as well as at 96 h (Fig. 4d). The profound changes in the phenotype of the liver shown here after two successive doses of APAP are likely to influence the fate of subsequent APAP exposure. Importantly, there was no evidence from analysis of intrahepatic albumin expression for a global loss of hepatocytes during the process of adaptation to APAP (Fig. 4e). Together with the ALT and histopathology data (Fig. 1), these observations indicate that although there is evidence of significant injury at 48 h, with key protein changes, albumin, a classical marker of liver function, remains invariant.

To look at the level of redundancy in the process of autoprotection to APAP, we then investigated the role of one of the key processes identified by pathway analysis, as described above, namely the ‘NFE2L2-mediated Oxidative Stress Response’. Nuclear factor erythroid 2-related factor 2 (NFE2L2; also known as NRF2) is a transcription factor that has been shown to play a vital role in the cytoprotective response against the type of oxidative and chemical stresses elicited by APAP^15^,^16^,^17^,^18^,^19^. We therefore employed a genetic strategy to test the hypothesis that interference with such a key pathway would affect the outcome of the adaptive response. The effect of pre-treatment with APAP on the ability of NFE2L2^+/+^ and NFE2L2^−/−^ mice to withstand a toxic challenge was investigated. All NFE2L2^+/+^ mice pre-treated with APAP survived a toxic challenge of APAP, whereas only 50% of NFE2L2^+/+^ mice which were not APAP-pre-treated survived (Fig. 4f). Amongst the NFE2L2^−/−^ mice, whilst none of the animals which were not APAP-pre-treated survived, 50% of animals which were APAP-pre-treated survived, despite NFE2L2 deletion. The effect of APAP pre-treatment on the ability of NFE2L2^+/+^ and NFE2L2^−/−^ mice to withstand a toxic challenge is summarised (Fig. 4f), and shows that in both wild type and NFE2L2^−/−^ mice, APAP pre-treatment increases survival upon toxic challenge, i.e. in the absence of a key liver defence pathway, adaptation still occurs. This clearly demonstrates for the first time that it is unlikely that a single gene or pathway underlies the complexities of the adaptive process to APAP exposure.

**Figure 5:**
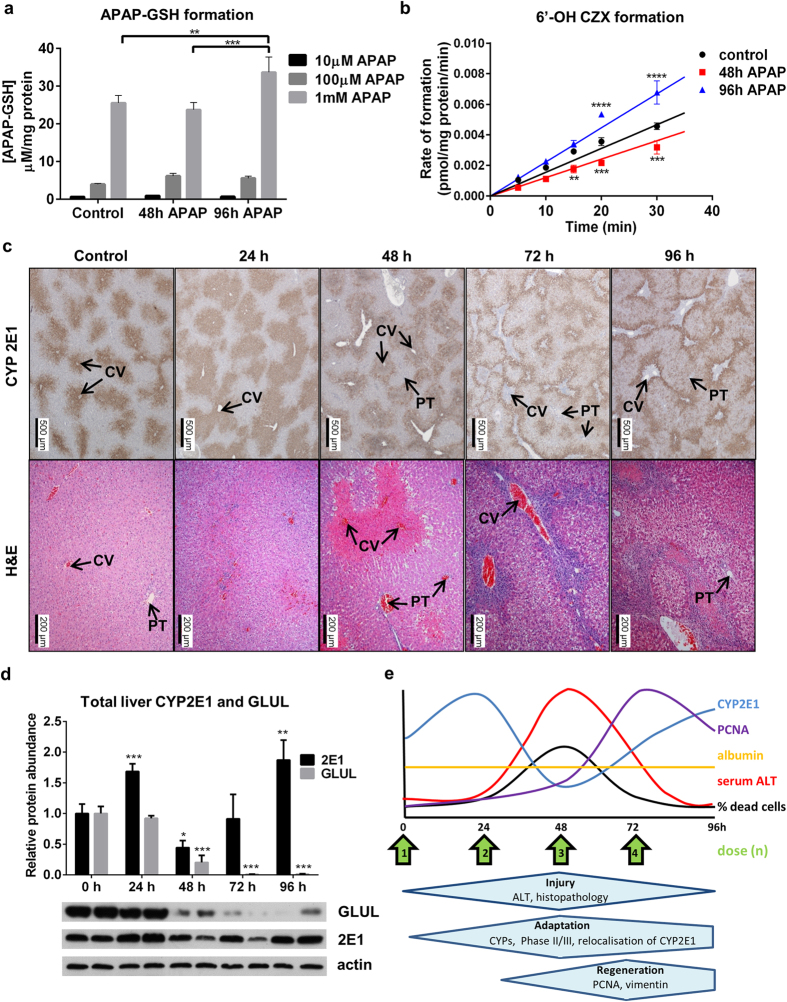
Rat liver CYP2E1 activity and localisation changes in response to repeat acetaminophen exposure. Microsomal formation of (**a**) APAP-GSH and (**b**) 6′-OH chlorzoxazone in animals which were either vehicle control treated, or repeat dosed with or without toxicity. Microsomal CYP2E1 activity is reduced in toxic (48 h treated, red squares) group and increased in non-toxic (96h treated, blue triangles) group compared to control (black circles). APAP metabolism is significantly higher in non-toxic (96h treated) group (**p < 0.01, ***p < 0.001, ****p < 0.0001). Best fit curves for each group (solid lines) were modelled using literature values for K_m_ and V_max_. (**c**) Top panel shows representative IHC staining for CYP2E1 across the timecourse; bottom panel shows H&E stain. CV indicates central vein, while PT indicates the portal triad. CYP2E1 redistributes markedly as the timecourse progresses. (**d**) Densitometric analysis of total liver GLUL, CYP2E1 and actin detected by western blot, showing preferential restitution of CYP2E1 abundance over the similarly centrilobular glutamine synthetase (*p < 0.05, **p < 0.01, ***p < 0.001). (**e**) Graphical summary of the model.

In fact, when we looked in greater detail at another of the key changes visualized in our proteomic analysis, we determined that the process of autoprotection is yet more complicated than straightforward changes in the expression of a specific protein. Our proteomic data for CYP2E1 (Fig. 4a,b) looked to be particularly pertinent in the process of adaptation to APAP, as it shows a loss of the pre-eminent metabolising enzyme responsible for formation of the toxic metabolite of APAP (NAPQI). We therefore looked at the metabolism of both APAP (Fig. 5a) and the CYP2E1 probe substrate chlorzoxazone (Fig. 5b) in our rat model. Whilst overall APAP metabolism was significantly increased at 96 h (Fig. 5a), specific CYP2E1 activity was reduced at 48 h and increased at 96 h (Fig. 5b). Relative CYP2E1 activity was 0.78 at 48 h, and 1.43 at 96 h, compared to control (arbitrary value of 1). These findings, which mirrored our proteomic data for CYP2E1, indicated the potential for an increase in the generation of NAPQI *in vivo*, which was difficult to resolve with the observed process of adaptation to repeated exposure.

We therefore employed immunohistochemical techniques to further probe the expression of CYP2E1. In fact, CYP2E1 is shown to undergo a profound change in its distribution within the liver during the process of adaptation to APAP (Fig. 5c). Basally, CYP2E1 is mainly expressed in centrilobular regions. Upon APAP challenge, CYP2E1 staining becomes diffuse and extends into midzonal regions. Two doses produce acute centrilobular necrosis, with markedly reduced CYP2E1 staining in centrilobular regions. At later time-points, staining extends diffusely into periportal regions which are CYP2E1-negative in control animals. This is clear evidence that the process of adaptation to APAP is yet more complex than wide-scale changes in the hepatoproteome, and may also involve a regional reprogramming of gene expression in a key step in APAP metabolism. This would not be detected if protein abundance alone was measured (Fig. 5d), which shows overall loss and re-establishment of liver CYP2E1, as predicted from the proteomic data (Fig. 4a,b). This re-direction of phase I metabolism towards the periportal areas of the liver, where the levels of the reducing buffer GSH are highest, is likely to be hepatoprotective. Critically, when we looked at expression of another protein which is highly abundant in the centrilobular region, glutamine synthetase (GLUL) we did not see the same change (Fig. 5d), showing some degree of selectivity in the restoration of adaptive liver function. This is the first time that a regio-spatial control of expression of a gene, specifically with respect to hepatic APAP autoprotection, and generally as part of a response to a pharmaceutical agent, has been shown. A graphical summary is presented in Fig. 5e and shows the evolution of key aspects of the adaptive process in this model.

## Discussion

Drug induced liver injury (DILI) is a major problem both in the clinic and for the pharmaceutical industry. Our relative lack of understanding of the physiological and toxicological mechanisms involved can lead to the loss of potentially effective drugs during development. APAP poisoning is itself also a significant clinical problem^1^,^2^. Furthermore, APAP is probably the best characterised hepatotoxin in pre-clinical models, and therefore provides a means to interrogate the various processes of adaptation and regeneration in the liver, which are also relevant to man and may be applicable to other drugs associated with DILI.

In our model of APAP autoprotection, significant hepatotoxicity was only observed at a dose of 1500 mg/kg, confirming that the rat is relatively resistant to APAP toxicity as reported earlier^20^,^21^,^22^. While hepatotoxicity was evident at 48 h (35% cell death, p < 0.001, data not shown), significantly less or no necrosis was observed at 72 and 96 h, despite repeated exposure to APAP. Liver function was maintained at these points, as assessed by serum transaminases and albumin synthesis. These observations demonstrate an adaptive response in this model, protecting the liver from further injury, and this adaptation involves changes in a very large proportion of the expressed hepatoproteome.

Our data during the early phase of the development of the model, confirms previous studies which have shown that hepatic CYP2E1, the predominant enzyme involved in APAP metabolism and CYP1A2, which also plays a minor role in APAP metabolism^23^,^24^, are depleted, whilst the rate-limiting enzyme involved in the formation of the protective tripeptide GSH, i.e. glutamate-cysteine ligase regulatory subunit (GCLC)^6^,^25^, is increased, after pre-treatment with APAP. These proteins are likely to contribute to protection from toxicity, by reducing the CYP-catalysed bioactivation of APAP to NAPQI, and by increasing detoxification of NAPQI through enhanced synthesis of GSH. However, in this study, which is the first to formally and quantitatively assess over one thousand liver proteins from an *in vivo* model during adaptation to chemical exposure, we can now see that these changes are only one small part of a greatly altered and dynamic phenotype.

The change in the hepatoproteome may enable an adaptation that prevents toxicity from subsequent doses of APAP and maintains normal liver function despite repeated exposure to a toxic dose. Although Fig. 3, showing the top 25 most perturbed canonical pathways, indicates widespread loss of protein abundance, particularly at the peak of toxicity, a number of pathways showed increased abundance, including ‘*acute phase response signalling’*, ‘*NFE2L2-mediated oxidative stress response’* and ‘*glutathione-mediated detoxification’* which are all well-characterised reactions to toxic injury. We therefore employed a conventional reductionist approach to examine in greater detail the role of transcription factor NFE2L2 in the process of adaptation. Whilst NFE2L2^−/−^ mice showed a greater susceptibility to APAP toxicity compared to wild types, after pretreatment with APAP an adaptive response was still evident in the absence of NFE2L2, albeit to a lesser degree. NFE2L2-mediated transcription is therefore not the only mechanism of adaptation to repeated toxic insult in this model, and provides a demonstration that the process of adaptation is more wide-ranging than previously thought.

Beyond confirming existing knowledge that several CYPs relevant to APAP metabolism are down-regulated upon repeat APAP exposure, we have shown that liver tissue from repeat dosed rats shows a significantly reduced abundance of proteins across all phases of drug metabolism (Fig. 4a–d). We hypothesise that this reduction in expression represents a key facet of adaptation, and provides an environment which facilitates compensatory hyperplastic activity, preserving critical function. At the peak of toxicity, the mean of all detected CYPs is reduced to approximately 60% of control values. This change in phenotype is likely to be, at least partly, a consequence of the changing cell population in the liver over time. The initial toxic insult clearly destroys vulnerable hepatocytes at the centre of the lobule. CYP enzymes show predominantly centrilobular expression, and the differential expression of CYPs in intact zones may account for the overall change seen at 48 h. Nevertheless, a number of CYPs are pan-zonally expressed in rat liver^26^, and of the ones that are also identified in the present work (1A, 2B, 2C6 and 2C7, a close homologue of the pan-zonal CYPEtOH2 enzyme^27^,^28^,^29^,^30^), all are significantly less abundant at 48 h, dropping to around 30% of control (with the exception of 2C7 which is not significantly changed). This suggests an active change in the phenotype of intact cells, as has been previously postulated, indicating a global dedifferentiation resulting in decreased liver-specific protein expression as part of an adaptive response to injury^31^,^32^, rather than a passive destruction of CYP-rich tissue.

To further elucidate the complex mechanism of adaptation, we focused on CYP2E1 activity and localisation in response to repeat APAP exposure. Consistent with proteomic data obtained for CYP2E1 abundance, microsomal activity assays show a loss of activity at 48h when acute injury is seen. Despite continued APAP exposure, however, at 96h a rebound of both expression and activity of CYP2E1 is seen (Fig. 5b,d). This is not consistent with the fall in toxicity observed after 48 h (Fig. 1). We therefore examined the tissue histologically, and identified a diffuse redistribution of CYP2E1 into CYP2E1-negative regions (Fig. 5c). Importantly, the loss and restoration of total liver 2E1 was not observed for another similarly perivenous enzyme, glutamine synthetase (Fig. 5d), suggesting that this process is selective. We hypothesise from these observations that, as a defence mechanism, diffuse expression of CYP2E1 may prevent the intracellular threshold of NAPQI toxicity being reached, thereby avoiding the initiation of further waves of cell death at later timepoints. Furthermore, in the regions where NAPQI is generated after redistribution of CYP2E1 expression, there are known to be higher intracellular concentrations of the reducing buffer GSH^33^. This phenomenon of CYP2E1 redistribution as an adaptive response has only been seen after treatment with carbon tetrachloride^34^ and ethanol^35^, but has not been detected previously after treatment with any pharmaceutical compound.

Proliferating liver demonstrates enhanced resistance to toxicity^4^,^25^,^36^, but the precise mechanism by which this occurs is unclear. Consistent with the findings presented here, rats which have been pretreated with APAP show signs of a regenerative hepatocyte response, determined through expression of PCNA, upon a second toxic challenge^25^. It has been shown that these new hepatocytes have a greater capacity for GSH production, allowing regenerating liver to detoxify NAPQI more efficiently^25^, but this hypothesis needs to be investigated in our model. Furthermore, a single dose of APAP has been shown to induce the expression of the transporter protein ABCC4 in proliferating hepatocytes, peaking at 48 h after dosing^6^. A number of transporters were detected in the current work, and of those that were changed, all were decreased in abundance at 48 h and onwards. A list of the transporters can be found in Supplementary Table 4. This may demonstrate a change in the liver phenotype away from drug metabolism and towards self-preservation and regeneration. A single dose control experiment was also performed in our study, and this showed a similar profile of toxicity to the repeat dose group (Supplementary Fig. 1). When experimental animals were given subsequent exposures, this toxicity was not exacerbated, indicating that a single exposure is all that is required to initiate liver adaptive processes.

How these observations relate to the likely hepatic changes seen during chronic dosing in humans will be a key next step in this work. The identification of accessible translational biomarkers that can be used in rodents and man^37^,^38^ will be necessary to examine whether this process occurs in man at therapeutic doses of APAP, as well as with other drugs than can cause DILI.

## Materials and Methods

### Materials

8-plex iTRAQ protein labelling kit/reagents were from AB Sciex (Framingham, MA). Sequencing grade trypsin was from Promega UK (Southampton, UK). PCNA, GLUL and NQO1 antibodies were from Abcam (Cambridge, UK; cat. no. ab29, ab49873 and ab2346 respectively). VIM antibody was from Sigma (St. Louis, MO; cat. no. V6389). GSTP1 antibody was from Enzo Life Sciences (Farmingdale, NY; msa-102). CYP2E1 antibody was kindly provided by Magnus Ingelman-Sundberg, Karolinska Institute, Sweden. Parent drugs for LC-MS analysis were obtained from Sigma-Aldrich (Gillingham, UK), internal standards from Toronto Research Chemicals (Toronto, Canada) and Alsachim (Illkirch-Graffenstaden, France). Metabolites were obtained from the same sources. All other reagents were of analytical grade and quality and purchased from Sigma (St. Louis, MO).

### Animals

Male Crl:WI (Han) rats and Crl:CD1 (ICR) mice (6–8 weeks) from Charles River Laboratories (Lyon, France) were acclimatised for 1 week. Six animals were housed per cage, on a 12 h light/dark cycle, at constant temperature (22 ± 2 °C). Standard food and tap water were provided *ad libitum*. Care of animals was undertaken in compliance with the European Community Directive 86/609/CEE for the use of laboratory animals and with the Autonomous Catalan law (Decret 214/1997). All experimental procedures were approved by the Almirall Ethics Committee.

### Study design

APAP was dissolved in vehicle (0.5% methylcellulose and 0.1% Tween 80 in distilled water, 10 ml/kg) and administered by oral (po) gavage without prior fasting. Formulations were prepared daily. Some animals (n = 6) received a single dose of APAP or vehicle at 0 h (for 2 or 24 h). Other animals (n = 6) received subsequent administrations every 24 h of APAP or vehicle for 24, 48 or 72 h and were sacrificed 24 h after last administration. Mice received 250, 500 or 750 mg/kg of APAP; rats received 500, 1000 or 1500 mg/kg of APAP. Based on clinical chemistry and histopathology analysis of liver injury, the rat 1500 mg/kg group was taken forward for further analysis. Terminal blood samples without previous fasting were collected from the retroorbital plexus under isoflurane anaesthesia (4% induction, 1.5–3% maintenance). Blood samples were centrifuged for 10 min at 3000 rpm, serum collected and stored at −80 °C. Immediately after blood collection, animals were exsanguinated by cutting the abdominal aorta under isoflurane anaesthesia.

### Toxicological assessment

A Synchron Clinical System cx7® (Beckman, Brea, CA) was used to determine ALT (IU/L) and AST (IU/L). Samples of liver were frozen in liquid nitrogen before storage at −80 °C. The hepatic median lobe was formalin-fixed and embedded in paraffin, sectioned and stained with haematoxylin and eosin (H&E) for histological blind examination under light microscopy. Percentage of live cells was assessed in a complete section from each animal, and data expressed as mean ± standard error of mean (SEM).

### NFE2L2^(−/−)^ study

All experiments were undertaken in accordance with criteria outlined in a licence granted under the Animals (Scientific Procedures) Act 1986, and approved by the Animal Ethics Committee of the University of Liverpool. Generation of the NFE2L2 knockout mouse and genotyping of progeny have been described elsewhere^15^,^16^. Non-fasted male littermate NFE2L2^(+/+)^ and NFE2L2^(−/−)^ mice (C57BL6J background, 10–12 weeks of age) were used throughout the study. Mice were housed between 19 °C–23 °C, on a 12 h light/dark cycle, and given access to food and water *ad libitum*. Dosing began at 10 am each day and APAP was freshly prepared in warmed saline (0.9%). Pilot dose ranging studies confirmed the dose dependent nature of APAP-induced hepatotoxicity. In order to explore both the changes occurring during the pre-treatment phase and the effects of pre-treatment on susceptibility to a toxic challenge, two independent groups of mice were used:

NFE2L2^(+/+)^ and NFE2L2^(−/−)^ mice received increasing daily doses of APAP (2 × 150 mg/kg, 2 × 300 mg/kg, 2 × 450 mg/kg, 2 × 600 mg/kg, *i.p.*) over 8 days, or vehicle control (*n* = 4). On day 9 mice were challenged with 1000 mg/kg APAP (*i.p.)* or vehicle (*n* = 4–8). Mice were culled 5 h after the final dose by exposure to a rising concentration of CO_2_ followed by cervical dislocation.

### iTRAQ labelling and mass spectrometric analysis of liver homogenates

Rat liver samples (n = 4 animals per time-point, 1500 mg/kg group, ~100 mg wet weight) were homogenised in 0.5 M triethylammonium bicarbonate/0.1% SDS using a Mixer Mill 220 (Retsch, Haan, Germany), and centrifuged at 14000 g for 10 min. iTRAQ tagging and analysis was performed as described previously^39^.

### iTRAQ protein identification and statistical analyses

Liver samples from rats treated with APAP or vehicle control were analysed across four iTRAQ runs. Data analysis was performed using ProteinPilot (Version 3, Life Technologies, Paisley, UK). The SwissProt database was searched with a confidence interval of 95%, and screened in reverse to facilitate false discovery rate analysis. Proteins identified from peptides with >95% confidence and global false discovery rate of <1% were included in the statistical analysis (1169 proteins).

Mean fold changes were calculated using the limma package within the R programming environment (Team, 2005) and analysis conducted on the logged fold-change values. Unadjusted (raw) p values and p values following FDR correction for multiple testing were determined.

### Ontology and pathway analysis

Pathway analysis was performed using Ingenuity Pathway Analysis (Qiagen, Venlo, Netherlands). Ingenuity successfully mapped 1163/1169 proteins to pathways. At each timepoint, a ‘Core Analysis’ was performed on all proteins that were differentially expressed compared to control animals (raw p < 0.05) using the ‘Ingenuity Knowledge Base (Gene Only) Background’. The canonical pathways that were statistically significantly altered at each timepoint were compared using the ‘Comparison Analysis’ function.

### Western immunoblotting

Buffered homogenates of standardised protein concentration (n = 4) were run on polyacrylamide gel, transferred onto nitrocellulose membrane (GE Healthcare, Little Chalfont, UK) and visualised using Western Lightning Plus ECL (Perkin Elmer, Waltham, MA). Proteins were normalised to actin. For GLUL, equal protein loading was confirmed using a Ponceau S stain.

### Enzyme activity

Microsomes were prepared from livers of animals assigned to 2 h vehicle control, 48 h APAP (repeat exposure with toxicity) or 96 h APAP (repeat exposure, no toxicity) groups by homogenisation and ultracentrifugation, quantified using the Lowry method^40^, snap frozen and stored at −80 °C until required. Microsomes from individual animals were incubated with either APAP (1 mM, 100 uM or 10 uM for up to 4 h) or the CYP2E1 probe chlorzoxazone (1 uM for up to 90 mins) at 37 °C in a shaking water bath. Reactions were stopped with the addition of equal volumes of ice cold acetonitrile containing evaporation standard fluconazole and stored at −80 °C until analysis by HPLC-MS.

### Analysis by HPLC-MS/MS

Test samples were treated with acetonitrile, to remove matrix-based interferences. They were diluted with water prior to analysis by LC-MS/MS on a Sciex API 4000 (Warrington, UK) equipped with a Turbo V™ electrospray source (ESI). The gradients were based on mobile phases containing 0.1% v/v formic acid in both water (A) and acetonitrile (B).

### Measurement of chlorzoxazone and its putative major metabolite, 6-hydroxy chlorzoxazone

The separation was performed on a 2.7 μM Halo® C18 column (50 × 2.1 mm ID) obtained from HiChrom (Reading, UK), and at a temperature of 40 °C and a flow-rate of 0.6 mL min^−1^. The following gradient was used: 0 min 5% B, 0.5 min 5% B, 1.5 min 95% B, 2 min 95% B, 2.1 min 5% B and 2.6 min 5% B. D^4^-diclofenac was employed as the internal standard. The MS was operated in negative ion mode.

### Measurement of APAP and its major metabolites

Separations were performed on a 2.6 μm Kinetex® XB-C18 column (50 × 2.1 mm ID) obtained from Phenomenex (Macclesfield, UK), at a temperature of 40 °C and a flow-rate of 0.5 mL min^−1^. The following gradient was used: 0 min 0% B, 0.3 min 0% B then 2.3 min 50% B. The column was flushed with 100% B, and then returned to 0% B using a flow-rate of 0.7 mL min^−1^, giving a programmed cycle time of 4.2 minutes. A panel of deuterated internal standards was employed. The MS was operated in negative ion mode for measuring all but one of the putative major metabolites and high concentrations of APAP. It was operated in positive ion mode for measuring the remaining metabolite and low concentrations of APAP.

### CYP2E1 localisation via immunohistochemistry

Immunohistochemical staining for CYP2E1 was performed on formalin-fixed, paraffin-embedded liver sections of 3 μm. Sections were mounted on poly-L-lysine coated slides, air-dried, deparaffinized and rehydrated. Slides were incubated with rabbit anti CYP2E1 antibody in 20% NGS/TBS-T. Secondary antibody was SignalStain® Boost IHC Detection Reagent (HRP, rabbit; Cell Signalling Technology, Beverly, MA, USA). The reaction was developed using 3′, 3′-diaminobenzidine tetrahydrochloride. Sections were counterstained with Harris haematoxylin.

### Data evaluation and statistical analysis

Clinical chemistry data are expressed as mean ± SEM (n = 6). Serum ALT and AST data for vehicle- and APAP-treated animals were compared to time-matched controls by one-way ANOVA with Tukey’s post-hoc test using GraphPad PRISM (version 6.03 for Windows, GraphPad Software, San Diego, CA). Western immunoblotting data were analysed using a one-way ANOVA with Dunnetts post-hoc test. Microsomal data were analysed using an ordinary two-way ANOVA with Dunnett’s post-hoc test.

Literature estimates for K_m_ are reported to be 30–300 times larger^41^,^42^ than the CZX concentration considered (1 uM) so we were able to adopt linear kinetics for CYP2E1 activity (enzyme velocity = α[S], where [S] = CZX concentration and α = V_max_/K_m_) instead of the full nonlinear Michaelis-Menten form. Solving the resulting first order ordinary differential equation then yields the following expression for 6′-OH CZX formation versus time (using notation [P](t) to denote concentration at time t (min)): [P](t) = [S](0)(1 − exp(−αt/V)), where [S](0) = initial CZX concentration (1 uM) and V is the sample volume (0.02 ml). We used a Levenberg Marquardt (non-linear regression) algorithm to then find best fit values for α = V_max_/K_m_ for the control, 48 and 96 hour cases. Note that, in each case, our estimates for α = V_max_/K_m_ were found to lie within acceptable literature ranges. Under the assumption that enzyme-substrate binding affinity (1/K_m_) is unaffected by microsomal conditions, ratios of α estimates were then used to compare relative enzyme activities.

## Additional Information

**How to cite this article**: Eakins, R. *et al.* Adaptation to acetaminophen exposure elicits major changes in expression and distribution of the hepatic proteome. *Sci. Rep.* **5**, 16423; doi: 10.1038/srep16423 (2015).

## Supplementary Material

Supplementary Information

## Figures and Tables

**Table 1:** Relative changes in protein abundance in rat liver in response to repeat acetaminophen exposure.

Timepoint (h)	Doses administered	Proteins increased abundance (p < 0.05)	Proteins decreased abundance (p < 0.05)	Total	Percentage of quantifiable proteome
24	1	30	43	73	6.24
48	2	116	199	315	26.95
72	3	111	145	256	21.90
96	4	86	125	211	18.05

The number of proteins that were increased or decreased in abundance in rat liver at each timepoint compared to control animals is indicated. The total number of changed proteins is expressed as a percentage of the total number of proteins quantified in the analysis (1169).
