# Supplementary material for: Adaptation to acetaminophen exposure elicits major changes in expression and distribution of the hepatic proteome
Source: Sci Rep. 2015 Nov 26;5:16423. doi: 10.1038/srep16423 (PMC4660393; doi:10.1038/srep16423)
Supplement: Supplementary Information [file srep16423-s1.pdf]

Adaptation to acetaminophen exposure elicits major changes in expression and distribution of the hepatic proteome

Eakins R*[1], Walsh J*[1], Randle L*[2], Jenkins RE[1], Schuppe-Koistinen I[3], Rowe C[1], Starkey Lewis P[1], Vasieva O[4], Prats N[5], Auli M[5], Bayliss M[1], Webb S[1], Rees, JA[1], Kitteringham NR[1], Goldring CE[#1], Park BK[1].

[1]MRC Centre for Drug Safety Science, University of Liverpool, Liverpool L69 3GE, UK

[2]Liverpool John Moores University, Byrom Street, Liverpool, L3 3AF, UK

[3]Almirall S. A. R&D Centre, Barcelona, Spain

[4]Institute of Integrative Biology, University of Liverpool, Liverpool, L69 7ZL, UK

[5]AstraZeneca R&D, Innovative Medicines, Personalised Healthcare & Biomarkers, Translational Science Centre, Science for Life Laboratory, Solna, Sweden

*These authors contributed equally

[#]Corresponding author

Supplementary Table 1A: *iTRAQ-based proteomic comparison of liver proteins in vehicle control- and APAP-treated rats (24h timepoint).* Proteins with expression that was different (raw P < 0.05) between control and APAP-treated rats at 24h are listed. Mean expression values relative to a common pool are given for n=4 animals. Proteins are ordered according to the ratio between the 24h and control groups (lowest to highest) such that proteins with expression that was most markedly reduced at 24h appear at the top of the list.

[a]Average number of peptides used for quantification across the four individual iTRAQ runs.

[b]Uncorrected raw P value.

Uniprot Accession	Name	Peptides[a]	Vehicle control		24h		24h/ctrl	P-value[b]
			Mean	SD	Mean	SD	Ratio	
**Proteins reduced at 24h**								
**P49890**	Estrogen sulfotransferase, isoform 6	18	3.22	0.91	0.14	0.07	0.04	<0.001
**P05369**	Farnesyl pyrophosphate synthase	27	1.50	0.39	0.62	0.21	0.42	0.009
**O35760**	Isopentenyl-diphosphate Delta-isomerase 1	4	1.38	0.41	0.69	0.27	0.50	0.035
**O88813**	Long-chain-fatty-acid--CoA ligase 5	28	1.89	0.67	0.97	0.31	0.52	0.036
**Q9ES38**	Bile acyl-CoA synthetase	26	2.80	0.86	1.45	0.44	0.52	0.022
**P52847**	Sulfotransferase family cytosolic 1B member 1	16	1.97	0.42	1.06	0.43	0.54	0.025
**P36510**	UDP-glucuronosyltransferase 2A1	3	1.35	0.34	0.75	0.29	0.56	0.033
**P63174**	60S ribosomal protein L38	5	0.96	0.24	0.56	0.24	0.58	0.041
**Q9WUS0**	Adenylate kinase isoenzyme 4, mitochondrial	14	1.38	0.19	0.81	0.26	0.59	0.024
**Q4V8F9**	Hydroxysteroid dehydrogenase-like protein 2	9	0.95	0.24	0.56	0.16	0.59	0.041
**P15083**	Polymeric immunoglobulin receptor	16	1.20	0.24	0.73	0.10	0.61	0.007
**Q6AXX6**	Redox-regulatory protein PAMM	8	1.54	0.33	0.94	0.10	0.61	0.010
**P16970**	ATP-binding cassette sub-family D member 3	13	1.69	0.46	1.03	0.07	0.61	0.018
**P02692**	Fatty acid-binding protein, liver	69	1.94	0.49	1.18	0.12	0.61	0.017
**P62271**	40S ribosomal protein S18	13	1.05	0.27	0.66	0.20	0.63	0.043
**P09527**	Ras-related protein Rab-7a	11	0.96	0.11	0.61	0.09	0.63	0.003
**P00173**	Cytochrome b5	31	1.37	0.20	0.88	0.21	0.64	0.018
**P27605**	Hypoxanthine-guanine phosphoribosyltransferase	12	1.35	0.20	0.87	0.18	0.64	0.012
**Q7TQM4**	Sterol O-acyltransferase 2	5	1.32	0.32	0.87	0.15	0.66	0.034

**P29411**	GTP:AMP phosphotransferase, mitochondrial	7	1.41	0.24	0.94	0.28	0.66	0.049
**P54921**	Alpha-soluble NSF attachment protein	10	1.22	0.29	0.81	0.07	0.66	0.023
**P10867**	L-gulonolactone oxidase	18	2.00	0.36	1.35	0.29	0.67	0.030
**P97612**	Fatty-acid amide hydrolase 1	26	1.67	0.31	1.15	0.20	0.69	0.033
**P57093**	Phytanoyl-CoA dioxygenase, peroxisomal	10	1.36	0.22	0.95	0.21	0.70	0.040
**Q5XIM9**	T-complex protein 1 subunit beta	31	0.97	0.08	0.68	0.09	0.70	0.004
**P11507**	Sarcoplasmic/endoplasmic reticulum calcium ATPase 2	30	1.01	0.10	0.75	0.09	0.74	0.007
**P50237**	Sulfotransferase 1C1	27	1.95	0.15	1.44	0.30	0.74	0.029
**Q9WUW9**	Sulfotransferase 1C2A	5	1.20	0.17	0.89	0.09	0.74	0.015
**P04646**	60S ribosomal protein L35a	3	1.04	0.06	0.78	0.16	0.75	0.041
**O88941**	Mannosyl-oligosaccharide glucosidase	12	1.12	0.13	0.85	0.08	0.76	0.008
**Q68FP2**	Serum paraoxonase/lactonase 3	16	1.43	0.10	1.09	0.15	0.76	0.011
**P29314**	40S ribosomal protein S9	13	0.86	0.10	0.65	0.05	0.76	0.009
**P06214**	Delta-aminolevulinic acid dehydratase	35	1.50	0.26	1.15	0.08	0.76	0.027
**P97562**	Peroxisomal acyl-coenzyme A oxidase 2	38	1.45	0.15	1.13	0.15	0.78	0.029
**Q03336**	Regucalcin	45	1.98	0.18	1.54	0.25	0.78	0.030
**P85108**	Tubulin beta-2A chain	72	1.11	0.05	0.87	0.06	0.78	0.001
**P18445**	60S ribosomal protein L27a	9	0.86	0.08	0.68	0.08	0.79	0.023
**Q5XI32**	F-actin-capping protein subunit beta	11	1.03	0.11	0.82	0.12	0.80	0.045
**P61107**	Ras-related protein Rab-14	9	1.11	0.14	0.89	0.04	0.80	0.016
**Q497B0**	Omega-amidase NIT2	34	1.58	0.09	1.28	0.13	0.81	0.013
**P41562**	Isocitrate dehydrogenase [NADP] cytoplasmic	59	1.21	0.08	1.00	0.07	0.82	0.007
**Q920J4**	Thioredoxin-like protein 1	8	1.12	0.11	0.94	0.06	0.84	0.026
**Q68FS4**	Cytosol aminopeptidase	53	1.52	0.14	1.33	0.06	0.88	0.044
**Proteins increased at 24h**								
**P08541**	UDP-glucuronosyltransferase 2B2	37	0.46	0.87	2.34	0.85	5.05	0.020
**P02091**	Hemoglobin subunit beta-1	179	0.44	0.20	2.03	1.54	4.58	0.029
**P38918**	Aflatoxin B1 aldehyde reductase member 3	42	0.20	0.17	0.69	0.31	3.40	0.024
**P13221**	Aspartate aminotransferase, cytoplasmic	30	0.51	0.20	1.35	0.49	2.66	0.010
**Q62651**	Delta(3,5)-Delta(2,4)-dienoyl-CoA isomerase, mitochondrial	17	0.62	0.20	1.59	0.44	2.55	0.005
**O70199**	UDP-glucose 6-dehydrogenase	38	0.32	0.10	0.77	0.16	2.42	0.003
**P06866**	Haptoglobin	21	0.33	0.15	0.77	0.28	2.35	0.033
**O09171**	Betaine--homocysteine S-methyltransferase 1	123	1.06	0.39	2.49	1.05	2.35	0.028

**P09034**	Argininosuccinate synthase	95	0.70	0.51	1.62	0.48	2.32	0.045
**P08649**	Complement C4	30	0.52	0.34	1.20	0.15	2.31	0.046
**P23562**	Band 3 anion transport protein	17	0.90	0.23	2.02	0.98	2.24	0.033
**P27139**	Carbonic anhydrase 2	18	0.68	0.32	1.52	0.49	2.23	0.032
**P20059**	Hemopexin	24	0.45	0.12	0.98	0.25	2.16	0.007
**P13255**	Glycine N-methyltransferase	37	0.86	0.37	1.83	0.71	2.14	0.044
**Q68FT5**	Betaine--homocysteine S-methyltransferase 2	82	0.99	0.45	2.08	0.57	2.10	0.028
**P48508**	Glutamate--cysteine ligase regulatory subunit	9	0.68	0.26	1.39	0.40	2.05	0.041
**P05182**	Cytochrome P450 2E1	29	0.89	0.14	1.74	0.48	1.96	0.006
**Q6URK4**	Heterogeneous nuclear ribonucleoprotein A3	20	0.56	0.07	0.99	0.29	1.76	0.027
**Q63416**	Inter-alpha-trypsin inhibitor heavy chain H3	10	0.81	0.25	1.31	0.25	1.62	0.036
**P20817**	Cytochrome P450 4A14	23	1.06	0.19	1.71	0.45	1.61	0.030
**Q58FK9**	Kynurenine--oxoglutarate transaminase 3	23	1.01	0.27	1.61	0.18	1.59	0.011
**P13635**	Ceruloplasmin	19	0.67	0.20	1.05	0.07	1.57	0.031
**P25409**	Alanine aminotransferase 1	19	0.85	0.09	1.13	0.14	1.32	0.018
**Q63584**	Transmembrane emp24 domain-containing protein 10	11	0.90	0.07	1.17	0.21	1.31	0.038
**P38062**	Methionine aminopeptidase 2	2	0.87	0.12	1.11	0.16	1.28	0.049
**P21571**	ATP synthase-coupling factor 6, mitochondrial	13	0.93	0.09	1.17	0.17	1.25	0.045
**P85834**	Elongation factor Tu, mitochondrial	26	1.01	0.12	1.23	0.13	1.22	0.045
**P63086**	Mitogen-activated protein kinase 1	5	0.83	0.08	1.00	0.11	1.21	0.041
**Q9Z1W6**	Protein LYRIC	7	1.17	0.14	1.38	0.06	1.18	0.037
**P02770**	Serum albumin	193	0.97	0.04	1.10	0.08	1.13	0.026

Supplementary Table 1B: *iTRAQ-based proteomic comparison of liver proteins in vehicle control- and APAP-treated rats (48h timepoint).* Proteins with expression that was different (raw P < 0.05) between control and APAP-treated rats at 48h are listed. Mean expression values relative to a common pool are given for n=4 animals. Proteins are ordered according to the ratio between the 48h and control groups (lowest to highest) such that proteins with expression that was most markedly reduced at 48h appear at the top of the list.

[a]Average number of peptides used for quantification across the four individual iTRAQ runs.

[b]Uncorrected raw P value.

Uniprot Accession	Name	Peptides[a]	Vehicle control Mean	Vehicle control SD	48h Mean	48h SD	48h/ctrl Ratio	P-value[b]
**Proteins reduced at 48h**								
**P49890**	Estrogen sulfotransferase, isoform 6	18	3.22	0.91	0.27	0.17	0.08	0.001
**P05183**	Cytochrome P450 3A2	15	2.49	0.97	0.29	0.23	0.12	0.001
**P08683**	Cytochrome P450 2C11	51	2.53	0.52	0.38	0.23	0.15	0.001
**P09606**	Glutamine synthetase	17	2.73	0.78	0.47	0.30	0.17	0.011
**P08010**	Glutathione S-transferase Mu 2	70	1.42	0.86	0.31	0.15	0.22	0.022
**Q9ES38**	Bile acyl-CoA synthetase	26	2.80	0.86	0.62	0.24	0.22	0.001
**P02761**	Major urinary protein	22	2.27	0.42	0.52	0.23	0.23	<0.001
**P10867**	L-gulonolactone oxidase	18	2.00	0.36	0.48	0.09	0.24	<0.001
**P16232**	Corticosteroid 11-beta-dehydrogenase isozyme 1	21	2.55	0.83	0.62	0.31	0.24	0.006
**P02692**	Fatty acid-binding protein, liver	69	1.94	0.49	0.48	0.32	0.25	0.003
**P52847**	Sulfotransferase family cytosolic 1B member 1	16	1.97	0.42	0.51	0.15	0.26	<0.001
**Q8VHE9**	All-trans-retinol 13,14-reductase	10	1.54	0.84	0.40	0.20	0.26	0.009
**P55051**	Fatty acid-binding protein, brain	9	1.61	0.57	0.42	0.47	0.26	0.037
**P24470**	Cytochrome P450 2C23	25	1.53	0.32	0.41	0.23	0.26	0.004
**P13107**	Cytochrome P450 2B3	27	1.48	0.55	0.40	0.52	0.27	0.033
**P18163**	Long-chain-fatty-acid--CoA ligase 1	78	1.54	0.13	0.41	0.16	0.27	0.001
**P50237**	Sulfotransferase 1C1	27	1.95	0.15	0.53	0.20	0.27	<0.001
**P36365**	Dimethylaniline monooxygenase [N-oxide-forming] 1	13	2.34	0.94	0.64	0.17	0.27	0.002
**Q03336**	Regucalcin	45	1.98	0.18	0.55	0.06	0.28	<0.001
**O88813**	Long-chain-fatty-acid--CoA ligase 5	28	1.89	0.67	0.53	0.30	0.28	0.007
**Q64638**	UDP-glucuronosyltransferase 1-5	25	3.79	1.58	1.08	0.77	0.28	0.009
**P30839**	Fatty aldehyde dehydrogenase	36	1.74	0.55	0.50	0.08	0.29	0.001

**P17988**	Sulfotransferase 1A1	30	1.99	0.34	0.58	0.09	0.29	<0.001
**P05178**	Cytochrome P450 2C6	20	1.67	0.62	0.50	0.24	0.30	0.009
**P14141**	Carbonic anhydrase 3	70	1.99	1.30	0.59	0.26	0.30	0.046
**P09811**	Glycogen phosphorylase, liver form	76	1.40	0.26	0.43	0.13	0.31	0.001
**P00502**	Glutathione S-transferase alpha-1	51	1.57	1.21	0.50	0.35	0.32	0.050
**P12928**	Pyruvate kinase isozymes R/L	61	1.64	0.53	0.55	0.19	0.34	0.003
**Q64232**	Very-long-chain enoyl-CoA reductase	35	1.76	0.41	0.60	0.22	0.34	0.003
**O35077**	Glycerol-3-phosphate dehydrogenase [NAD+], cytoplasmic	39	1.45	0.42	0.51	0.07	0.35	0.001
**Q64611**	Cysteine sulfinic acid decarboxylase	25	1.81	0.70	0.64	0.15	0.35	0.003
**P05182**	Cytochrome P450 2E1	29	0.89	0.14	0.32	0.17	0.36	0.013
**P15865**	Histone H1.4	19	1.04	0.35	0.38	0.12	0.36	0.008
**P31210**	3-oxo-5-beta-steroid 4-dehydrogenase	51	1.07	0.22	0.39	0.30	0.36	0.014
**Q5U2Q3**	Ester hydrolase C11orf54 homolog	12	2.00	0.36	0.74	0.38	0.37	0.006
**Q497B0**	Omega-amidase NIT2	34	1.58	0.09	0.59	0.13	0.37	<0.001
**P04799**	Cytochrome P450 1A2	7	1.32	0.36	0.49	0.48	0.37	0.024
**Q6AYT9**	Acyl-coenzyme A synthetase ACSM5, mitochondrial	14	1.53	0.30	0.57	0.17	0.37	0.001
**Q07071**	Glucokinase regulatory protein	27	1.71	0.64	0.65	0.23	0.38	0.015
**P43278**	Histone H1.0	4	1.51	0.31	0.58	0.19	0.38	0.003
**P16638**	ATP-citrate synthase	65	1.93	0.71	0.74	0.12	0.38	0.003
**Q4KLP0**	Probable 2-oxoglutarate dehydrogenase E1 component DHKTD1, mitochondrial	35	1.59	0.56	0.61	0.22	0.39	0.006
**P15149**	Cytochrome P450 2A2	35	1.28	0.09	0.50	0.07	0.39	<0.001
**P97612**	Fatty-acid amide hydrolase 1	26	1.67	0.31	0.65	0.19	0.39	0.002
**P18757**	Cystathionine gamma-lyase	37	1.40	0.63	0.55	0.21	0.39	0.021
**P19112**	Fructose-1,6-bisphosphatase 1	72	1.71	0.38	0.67	0.16	0.39	0.002
**Q5PPL3**	Sterol-4-alpha-carboxylate 3-dehydrogenase, decarboxylating	20	1.74	0.46	0.69	0.25	0.40	0.008
**P05545**	Serine protease inhibitor A3K	34	1.50	0.50	0.60	0.28	0.40	0.016
**P00884**	Fructose-bisphosphate aldolase B	129	1.31	0.33	0.52	0.16	0.40	0.005
**Q64550**	UDP-glucuronosyltransferase 1-1	29	1.54	0.70	0.62	0.21	0.40	0.016
**P29147**	D-beta-hydroxybutyrate dehydrogenase, mitochondrial	42	1.40	0.23	0.56	0.18	0.40	0.002
**P33274**	Cytochrome P450 4F1	14	1.35	0.35	0.55	0.28	0.41	0.029
**P27867**	Sorbitol dehydrogenase	26	1.21	0.44	0.50	0.10	0.41	0.011

**Q02253**	Methylmalonate-semialdehyde dehydrogenase [acylating], mitochondrial	124	1.22	0.24	0.51	0.06	0.42	<0.001
**Q9Z339**	Glutathione S-transferase omega-1	10	1.42	0.40	0.59	0.32	0.42	0.025
**P55159**	Serum paraoxonase/arylesterase 1	17	1.68	0.55	0.71	0.06	0.42	0.003
**P17764**	Acetyl-CoA acetyltransferase, mitochondrial	60	1.28	0.07	0.54	0.26	0.42	0.014
**Q63357**	Myosin-Id	93	1.01	0.12	0.43	0.22	0.42	0.014
**Q4KLZ6**	Bifunctional ATP-dependent dihydroxyacetone kinase/FAD-AMP lyase (cyclizing)	64	1.70	0.39	0.72	0.23	0.43	0.005
**Q6AYT0**	Quinone oxidoreductase	9	1.37	0.36	0.59	0.21	0.43	0.017
**Q07523**	Hydroxyacid oxidase 2	8	1.99	0.34	0.87	0.24	0.44	0.002
**P16303**	Carboxylesterase 3	33	1.85	0.85	0.81	0.19	0.44	0.030
**P22791**	Hydroxymethylglutaryl-CoA synthase, mitochondrial	106	1.46	0.40	0.65	0.26	0.44	0.010
**Q62730**	Estradiol 17-beta-dehydrogenase 2	17	1.72	0.47	0.76	0.36	0.44	0.034
**P13803**	Electron transfer flavoprotein subunit alpha, mitochondrial	50	1.29	0.23	0.59	0.25	0.46	0.011
**Q63150**	Dihydropyrimidinase	29	1.41	0.17	0.64	0.32	0.46	0.020
**P24329**	Thiosulfate sulfurtransferase	41	1.33	0.20	0.61	0.08	0.46	<0.001
**P46953**	3-hydroxyanthranilate 3,4-dioxygenase	25	1.42	0.40	0.66	0.06	0.47	0.003
**Q6DGG1**	Alpha/beta hydrolase domain-containing protein 14B	12	1.36	0.25	0.64	0.14	0.47	0.002
**P57113**	Maleylacetoacetate isomerase	36	1.58	0.34	0.74	0.14	0.47	0.002
**P97532**	3-mercaptopyruvate sulfurtransferase	23	1.27	0.28	0.61	0.09	0.48	0.001
**Q5FVQ4**	Malectin	8	1.39	0.58	0.67	0.31	0.48	0.043
**P52759**	Ribonuclease UK114	51	1.29	0.41	0.62	0.23	0.48	0.035
**P02696**	Retinol-binding protein 1	13	1.29	0.23	0.62	0.15	0.48	0.003
**P22734**	Catechol O-methyltransferase	46	1.49	0.39	0.72	0.22	0.48	0.009
**P08011**	Microsomal glutathione S-transferase 1	25	1.57	0.62	0.76	0.34	0.48	0.044
**A0JPQ8**	Alkylglycerol monooxygenase	6	1.64	0.20	0.80	0.12	0.49	<0.001
**Q498D5**	Regulator of microtubule dynamics protein 2	8	1.34	0.14	0.65	0.21	0.49	0.003
**P30713**	Glutathione S-transferase theta-2	19	1.34	0.31	0.65	0.10	0.49	0.002
**Q9QZX8**	Solute carrier organic anion transporter family member 1B2	3	1.36	0.40	0.67	0.19	0.49	0.009

**Q7TP52**	Carboxymethylenebutenolidase homolog	16	1.79	0.45	0.88	0.32	0.49	0.018
**P27605**	Hypoxanthine-guanine phosphoribosyltransferase	12	1.35	0.20	0.66	0.12	0.49	0.001
**P25093**	Fumarylacetoacetase	48	1.45	0.12	0.71	0.12	0.49	<0.001
**Q02974**	Ketohexokinase	25	1.56	0.28	0.77	0.07	0.50	<0.001
**Q5BK17**	Iodotyrosine dehalogenase 1	4	1.75	0.28	0.87	0.22	0.50	0.004
**P57093**	Phytanoyl-CoA dioxygenase, peroxisomal	10	1.36	0.22	0.68	0.18	0.50	0.004
**Q66HG4**	Aldose 1-epimerase	5	1.37	0.18	0.70	0.27	0.51	0.008
**P46720**	Solute carrier organic anion transporter family member 1A1	4	1.41	0.30	0.72	0.10	0.51	0.003
**Q63060**	Glycerol kinase	20	1.68	0.28	0.87	0.19	0.51	0.002
**Q68FP2**	Serum paraoxonase/lactonase 3	16	1.43	0.10	0.74	0.11	0.51	<0.001
**P10760**	Adenosylhomocysteinase	54	1.29	0.20	0.67	0.25	0.51	0.011
**P23680**	Serum amyloid P-component	4	1.61	0.24	0.83	0.08	0.52	<0.001
**P23457**	3-alpha-hydroxysteroid dehydrogenase	48	1.51	0.36	0.78	0.33	0.52	0.048
**P10860**	Glutamate dehydrogenase 1, mitochondrial	118	1.63	0.34	0.84	0.15	0.52	0.005
**Q64581**	Cytochrome P450 3A18	8	1.50	0.57	0.78	0.17	0.52	0.019
**P06214**	Delta-aminolevulinic acid dehydratase	35	1.50	0.26	0.78	0.14	0.52	0.002
**B0BNE5**	S-formylglutathione hydrolase	23	1.32	0.33	0.69	0.25	0.52	0.020
**Q562C4**	Methyltransferase-like protein 7B	8	1.63	0.70	0.85	0.17	0.52	0.028
**P08461**	Dihydrolipoyllysine-residue acetyltransferase component of pyruvate dehydrogenase complex, mitochondrial	11	1.13	0.26	0.59	0.06	0.52	0.005
**P41562**	Isocitrate dehydrogenase [NADP] cytoplasmic	59	1.21	0.08	0.63	0.15	0.52	0.002
**Q63270**	Cytoplasmic aconitate hydratase	36	1.21	0.20	0.64	0.21	0.52	0.010
**P70712**	Kynureninase	19	1.41	0.32	0.74	0.13	0.52	0.005
**Q5I0M2**	Nicotinate-nucleotide pyrophosphorylase [carboxylating]	11	1.28	0.42	0.67	0.27	0.53	0.041
**P62959**	Histidine triad nucleotide-binding protein 1	8	1.59	0.36	0.84	0.20	0.53	0.008
**Q9WUS0**	Adenylate kinase isoenzyme 4, mitochondrial	14	1.38	0.19	0.73	0.38	0.53	0.026
**O89000**	Dihydropyrimidine dehydrogenase [NADP+]	12	1.36	0.32	0.72	0.32	0.53	0.030
**P00173**	Cytochrome b5	31	1.37	0.20	0.73	0.30	0.53	0.020
**P10868**	Guanidinoacetate N-methyltransferase	16	1.58	0.51	0.85	0.15	0.53	0.018
**Q6P6R2**	Dihydrolipoyl dehydrogenase, mitochondrial	25	1.16	0.23	0.62	0.23	0.54	0.023

**P11915**	Non-specific lipid-transfer protein	31	1.23	0.23	0.66	0.31	0.54	0.049
**Q03248**	Beta-ureidopropionase	30	1.21	0.25	0.66	0.11	0.54	0.005
**P11884**	Aldehyde dehydrogenase, mitochondrial	98	1.17	0.24	0.64	0.16	0.55	0.011
**P04762**	Catalase	160	1.35	0.36	0.74	0.27	0.55	0.038
**P0C2X9**	Delta-1-pyrroline-5-carboxylate dehydrogenase, mitochondrial	43	1.30	0.10	0.71	0.09	0.55	<0.001
**P11348**	Dihydropteridine reductase	27	1.29	0.44	0.71	0.15	0.55	0.039
**Q9WVK7**	Hydroxyacyl-coenzyme A dehydrogenase, mitochondrial	54	1.22	0.31	0.68	0.18	0.55	0.026
**P10634**	Cytochrome P450 2D26	57	1.53	0.31	0.85	0.25	0.56	0.022
**P12938**	Cytochrome P450 2D3	46	1.52	0.33	0.85	0.17	0.56	0.010
**P38652**	Phosphoglucomutase-1	58	1.05	0.17	0.59	0.13	0.56	0.007
**P51869**	Cytochrome P450 4F4	14	1.42	0.51	0.80	0.05	0.56	0.038
**P19643**	Amine oxidase [flavin-containing] B	29	1.30	0.32	0.74	0.03	0.56	0.003
**Q5FVQ8**	NLR family member X1	9	1.14	0.25	0.65	0.26	0.57	0.046
**P84817**	Mitochondrial fission 1 protein	2	1.28	0.25	0.74	0.09	0.58	0.004
**P07953**	6-phosphofructo-2-kinase/fructose-2,6-bisphosphatase 1	13	1.47	0.11	0.85	0.09	0.58	<0.001
**P97524**	Very long-chain acyl-CoA synthetase	34	1.53	0.35	0.89	0.25	0.58	0.021
**P07896**	Peroxisomal bifunctional enzyme	45	1.24	0.32	0.72	0.22	0.58	0.038
**Q5XIC0**	Enoyl-CoA delta isomerase 2, mitochondrial	14	1.20	0.24	0.71	0.24	0.59	0.046
**Q5HZY2**	GTP-binding protein SAR1b	13	1.22	0.23	0.72	0.11	0.59	0.004
**Q0VGK3**	Glycerate kinase	16	1.22	0.15	0.72	0.29	0.59	0.043
**P38718**	Mitochondrial pyruvate carrier 2/Brain protein 44	3	1.35	0.43	0.79	0.18	0.59	0.041
**P16617**	Phosphoglycerate kinase 1	54	1.09	0.24	0.65	0.19	0.59	0.031
**P00481**	Ornithine carbamoyltransferase, mitochondrial	57	1.30	0.19	0.77	0.12	0.59	0.003
**P81155**	Voltage-dependent anion-selective channel protein 2	10	1.09	0.22	0.65	0.24	0.59	0.039
**Q68FS4**	Cytosol aminopeptidase	53	1.52	0.14	0.90	0.19	0.59	0.003
**P48500**	Triosephosphate isomerase	33	1.17	0.32	0.69	0.22	0.59	0.040
**Q68FU3**	Electron transfer flavoprotein subunit beta	29	1.17	0.22	0.70	0.20	0.60	0.025
**P00507**	Aspartate aminotransferase, mitochondrial	73	1.07	0.15	0.64	0.19	0.60	0.016
**Q6UPE1**	Electron transfer flavoprotein-ubiquinone oxidoreductase, mitochondrial	36	1.12	0.12	0.67	0.07	0.60	0.001
**P51635**	Alcohol dehydrogenase [NADP+]	24	1.25	0.27	0.75	0.15	0.60	0.014

**P27364**	3 beta-hydroxysteroid dehydrogenase type 5	19	1.41	0.31	0.85	0.06	0.60	0.004
**Q6P6V0**	Glucose-6-phosphate isomerase	32	1.23	0.31	0.74	0.15	0.60	0.025
**Q9QZH8**	Arylacetamide deacetylase	6	1.64	0.55	0.99	0.15	0.60	0.034
**Q6AXX6**	Redox-regulatory protein PAMM	8	1.54	0.33	0.93	0.25	0.60	0.026
**P56574**	Isocitrate dehydrogenase [NADP], mitochondrial	27	1.10	0.24	0.67	0.18	0.61	0.037
**P49432**	Pyruvate dehydrogenase E1 component subunit beta, mitochondrial	16	1.20	0.30	0.73	0.20	0.61	0.034
**Q6I7R3**	Isochorismatase domain-containing protein 1	10	1.44	0.23	0.87	0.12	0.61	0.005
**P08503**	Medium-chain specific acyl-CoA dehydrogenase, mitochondrial	26	1.49	0.32	0.91	0.22	0.61	0.017
**P30904**	Macrophage migration inhibitory factor	22	1.31	0.21	0.80	0.19	0.61	0.018
**P41034**	Alpha-tocopherol transfer protein	16	1.28	0.15	0.78	0.03	0.61	<0.001
**Q562C9**	1,2-dihydroxy-3-keto-5-methylthiopentene dioxygenase	8	1.34	0.34	0.82	0.16	0.61	0.025
**P31044**	Phosphatidylethanolamine-binding protein 1	22	1.26	0.27	0.78	0.15	0.62	0.018
**P07153**	Dolichyl-diphosphooligosaccharide--protein glycosyltransferase subunit 1	56	1.08	0.27	0.67	0.21	0.62	0.049
**P05369**	Farnesyl pyrophosphate synthase	27	1.50	0.39	0.93	0.14	0.62	0.024
**P35738**	2-oxoisovalerate dehydrogenase subunit beta, mitochondrial	13	1.23	0.12	0.76	0.23	0.62	0.016
**Q5M875**	17-beta-hydroxysteroid dehydrogenase 13	22	1.09	0.17	0.68	0.10	0.62	0.005
**P50137**	Transketolase	80	1.15	0.20	0.72	0.16	0.62	0.016
**P11951**	Cytochrome c oxidase subunit 6C-2	4	1.03	0.20	0.64	0.26	0.63	0.050
**Q920P0**	L-xylulose reductase	12	1.44	0.45	0.91	0.05	0.63	0.028
**P20070**	NADH-cytochrome b5 reductase 3	26	1.62	0.42	1.02	0.32	0.63	0.043
**P51650**	Succinate-semialdehyde dehydrogenase, mitochondrial	10	1.21	0.32	0.76	0.06	0.63	0.019
**Q63276**	Bile acid-CoA:amino acid N-acyltransferase	57	1.25	0.27	0.79	0.20	0.63	0.026
**Q5PQT3**	Glycine N-acyltransferase	22	1.05	0.24	0.67	0.06	0.64	0.016
**Q9QYU4**	Thiomorpholine-carboxylate dehydrogenase	9	1.31	0.25	0.84	0.17	0.64	0.017
**P25235**	Dolichyl-diphosphooligosaccharide--protein glycosyltransferase subunit 2	46	1.18	0.28	0.76	0.15	0.64	0.028

**P85971**	6-phosphogluconolactonase	12	1.56	0.23	1.02	0.10	0.65	0.003
**P97562**	Peroxisomal acyl-coenzyme A oxidase 2	38	1.45	0.15	0.94	0.22	0.65	0.021
**P29266**	3-hydroxyisobutyrate dehydrogenase, mitochondrial	23	1.21	0.17	0.79	0.15	0.65	0.010
**Q1HCL7**	NAD kinase domain-containing protein 1	17	1.25	0.18	0.82	0.05	0.66	0.002
**Q923K9**	APOBEC1 complementation factor	10	1.09	0.26	0.72	0.16	0.66	0.040
**P18297**	Sepiapterin reductase	11	1.36	0.27	0.90	0.17	0.66	0.017
**P32089**	Tricarboxylate transport protein, mitochondrial	11	1.11	0.09	0.74	0.17	0.67	0.015
**Q6P7R8**	Estradiol 17-beta-dehydrogenase 12	6	1.15	0.20	0.77	0.10	0.67	0.013
**P07633**	Propionyl-CoA carboxylase beta chain, mitochondrial	23	1.21	0.18	0.81	0.17	0.67	0.017
**P85973**	Purine nucleoside phosphorylase	41	1.12	0.20	0.75	0.12	0.67	0.016
**P15999**	ATP synthase subunit alpha, mitochondrial	122	1.05	0.08	0.71	0.16	0.68	0.012
**O88767**	Protein DJ-1	16	1.21	0.20	0.82	0.16	0.68	0.025
**P14942**	Glutathione S-transferase alpha-4	12	0.99	0.12	0.67	0.12	0.68	0.008
**Q9JM53**	Apoptosis-inducing factor 1, mitochondrial	27	1.24	0.13	0.85	0.20	0.69	0.026
**P07872**	Peroxisomal acyl-coenzyme A oxidase 1	42	1.33	0.25	0.92	0.17	0.70	0.046
**Q923M1**	Mitochondrial peptide methionine sulfoxide reductase	3	1.19	0.24	0.84	0.12	0.70	0.024
**Q6AYQ8**	Acylpyruvase FAHD1, mitochondrial	10	1.15	0.22	0.81	0.11	0.71	0.039
**Q6P7Q4**	Lactoylglutathione lyase	8	1.19	0.21	0.85	0.16	0.71	0.046
**Q5XIH7**	Prohibitin-2	29	1.66	0.28	1.21	0.24	0.73	0.046
**P52873**	Pyruvate carboxylase, mitochondrial	134	1.16	0.13	0.86	0.14	0.74	0.018
**Q5M7T9**	Threonine synthase-like 2	6	1.26	0.23	0.94	0.11	0.74	0.037
**P12336**	Solute carrier family 2, facilitated glucose transporter member 2	5	1.16	0.21	0.86	0.09	0.74	0.038
**P46462**	Transitional endoplasmic reticulum ATPase	71	1.14	0.18	0.85	0.11	0.75	0.034
**Q64380**	Sarcosine dehydrogenase, mitochondrial	41	1.28	0.19	0.96	0.15	0.75	0.047
**Q8CG45**	Aflatoxin B1 aldehyde reductase member 2	22	1.11	0.05	0.84	0.07	0.75	0.001
**P97700**	Mitochondrial 2-oxoglutarate/malate carrier protein	7	1.15	0.19	0.87	0.05	0.75	0.016
**Q68FT1**	Ubiquinone biosynthesis protein COQ9, mitochondrial	9	1.01	0.06	0.77	0.08	0.76	0.004
**Q7TMA5**	Apolipoprotein B-100	29	1.09	0.21	0.83	0.08	0.76	0.047
**P97519**	Hydroxymethylglutaryl-CoA lyase, mitochondrial	14	0.99	0.09	0.77	0.14	0.78	0.036

**Q641Y0**	Dolichyl-diphosphooligosaccharide--protein glycosyltransferase 48 kDa subunit	16	1.18	0.12	0.92	0.13	0.78	0.032
**P80067**	Dipeptidyl peptidase 1	12	1.09	0.19	0.86	0.04	0.79	0.039
**Q9JLA3**	UDP-glucose:glycoprotein glucosyltransferase 1	28	0.97	0.06	0.78	0.06	0.80	0.003
**P26453**	Basigin	7	1.15	0.14	0.96	0.00	0.84	0.028
**Q05096**	Myosin-Ib	94	1.03	0.04	0.87	0.08	0.84	0.013
**P84903**	Stromal interaction molecule 1	5	1.03	0.03	0.91	0.09	0.88	0.047
**Proteins increased at 48h**								
**P42930**	Heat shock protein beta-1	10	0.12	0.11	2.87	1.41	23.49	<0.001
**Q07439**	Heat shock 70 kDa protein 1A/1B	56	0.21	0.20	2.77	1.50	13.39	0.002
**P38918**	Aflatoxin B1 aldehyde reductase member 3	42	0.20	0.17	1.55	0.68	7.60	0.003
**P01048**	T-kininogen 1	15	0.25	0.21	1.83	0.91	7.21	0.010
**P02764**	Alpha-1-acid glycoprotein	6	0.31	0.37	1.83	0.22	5.87	0.017
**P06866**	Haptoglobin	21	0.33	0.15	1.74	0.81	5.26	0.003
**P82995**	Heat shock protein HSP 90-alpha	74	0.47	0.29	2.00	0.82	4.25	0.006
**P05370**	Glucose-6-phosphate 1-dehydrogenase	15	0.38	0.22	1.63	0.36	4.24	0.004
**O70199**	UDP-glucose 6-dehydrogenase	38	0.32	0.10	1.28	0.34	4.04	0.001
**Q66HA8**	Heat shock protein 105 kDa	23	0.47	0.24	1.72	0.21	3.64	0.004
**P02680**	Fibrinogen gamma chain	17	0.54	0.24	1.86	0.65	3.43	0.008
**P20059**	Hemopexin	24	0.45	0.12	1.53	0.73	3.37	0.005
**P06762**	Heme oxygenase 1	5	0.57	0.28	1.82	0.76	3.21	0.027
**P06399**	Fibrinogen alpha chain	39	0.50	0.29	1.58	0.31	3.18	0.019
**P09006**	Serine protease inhibitor A3N	19	0.61	0.19	1.82	0.67	3.00	0.003
**P04961**	Proliferating cell nuclear antigen	4	0.41	0.07	1.21	0.37	2.97	0.001
**O89049**	Thioredoxin reductase 1, cytoplasmic	17	0.42	0.29	1.19	0.36	2.87	0.031
**P13383**	Nucleolin	21	0.47	0.15	1.33	0.13	2.87	<0.001
**P11980**	Pyruvate kinase isozymes M1/M2	19	0.44	0.09	1.23	0.38	2.81	0.002
**P17475**	Alpha-1-antiproteinase	39	0.47	0.18	1.31	0.57	2.77	0.012
**P62982**	Ubiquitin-40S ribosomal protein S27a	23	0.50	0.30	1.38	0.33	2.73	0.018
**P05982**	NAD(P)H dehydrogenase [quinone] 1	8	0.50	0.12	1.37	0.37	2.73	0.002
**P07150**	Annexin A1	12	0.55	0.23	1.47	0.49	2.70	0.015
**P05371**	Clusterin	6	0.56	0.31	1.52	0.52	2.70	0.041
**P04906**	Glutathione S-transferase P	12	0.26	0.22	0.70	0.41	2.69	0.045
**O35821**	Myb-binding protein 1A	15	0.53	0.17	1.40	0.25	2.63	0.002
**P14480**	Fibrinogen beta chain	25	0.52	0.24	1.36	0.55	2.63	0.026

**Q3B8Q1**	Nucleolar RNA helicase 2	7	0.48	0.20	1.26	0.26	2.62	0.005
**P13084**	Nucleophosmin	14	0.51	0.21	1.29	0.17	2.53	0.011
**P63018**	Heat shock cognate 71 kDa protein	106	0.60	0.12	1.51	0.35	2.50	0.001
**P06761**	78 kDa glucose-regulated protein	112	0.69	0.25	1.70	0.50	2.47	0.008
**P52631**	Signal transducer and activator of transcription 3	10	0.64	0.30	1.57	0.46	2.45	0.011
**P08430**	UDP-glucuronosyltransferase 1-6	40	0.58	0.23	1.37	0.48	2.34	0.017
**D4A4T9**	Cysteine and histidine-rich domain-containing protein 1	3	0.86	0.23	2.00	1.03	2.32	0.046
**Q62812**	Myosin-9	161	0.57	0.26	1.31	0.35	2.30	0.018
**P63029**	Translationally-controlled tumor protein	9	0.69	0.20	1.52	0.27	2.20	0.004
**P05765**	40S ribosomal protein S21	10	0.55	0.32	1.21	0.25	2.20	0.032
**P62961**	Nuclease-sensitive element-binding protein 1	22	0.71	0.33	1.55	0.19	2.17	0.025
**P05197**	Elongation factor 2	70	0.71	0.15	1.55	0.27	2.17	0.001
**O35814**	Stress-induced-phosphoprotein 1	24	0.72	0.16	1.54	0.71	2.15	0.031
**P34058**	Heat shock protein HSP 90-beta	80	0.77	0.18	1.65	0.41	2.14	0.005
**B0BNA5**	Coactosin-like protein	3	0.60	0.19	1.27	0.44	2.11	0.022
**Q63525**	Nuclear migration protein nudC	9	0.51	0.16	1.05	0.42	2.05	0.035
**P31000**	Vimentin	32	0.51	0.15	1.03	0.26	2.04	0.012
**Q62651**	Delta(3,5)-Delta(2,4)-dienoyl-CoA isomerase, mitochondrial	17	0.62	0.20	1.26	0.50	2.03	0.026
**O35763**	Moesin	19	0.58	0.12	1.18	0.14	2.03	0.001
**Q6AYC2**	Immunity-related GTPase family M protein	3	0.71	0.25	1.43	0.29	2.02	0.017
**P50503**	Hsc70-interacting protein	14	0.69	0.32	1.39	0.50	2.02	0.047
**P13221**	Aspartate aminotransferase, cytoplasmic	30	0.51	0.20	1.01	0.37	1.99	0.043
**Q9JJ19**	Na(+)/H(+) exchange regulatory cofactor NHE-RF1	13	0.70	0.17	1.37	0.43	1.95	0.021
**Q6URK4**	Heterogeneous nuclear ribonucleoprotein A3	20	0.56	0.07	1.08	0.26	1.91	0.004
**Q1JU68**	Eukaryotic translation initiation factor 3 subunit A	23	0.66	0.17	1.26	0.26	1.91	0.007
**Q07936**	Annexin A2	15	0.61	0.20	1.13	0.38	1.87	0.036
**P26772**	10 kDa heat shock protein, mitochondrial	13	0.72	0.29	1.34	0.12	1.87	0.016
**Q9Z2G8**	Nucleosome assembly protein 1-like 1	5	0.72	0.14	1.35	0.24	1.87	0.005
**Q5M9G3**	Caprin-1	4	0.88	0.24	1.65	0.58	1.87	0.035
**P45592**	Cofilin-1	14	0.65	0.06	1.21	0.16	1.86	<0.001
**P70619**	Glutathione reductase (Fragment)	8	0.68	0.17	1.25	0.29	1.85	0.014
**O35987**	NSFL1 cofactor p47	14	0.71	0.37	1.29	0.21	1.82	0.036
**Q5BJY9**	Keratin, type I cytoskeletal 18	82	0.77	0.29	1.35	0.23	1.76	0.040

**P69897**	Tubulin beta-5 chain	75	0.53	0.17	0.92	0.20	1.75	0.027
**Q63617**	Hypoxia up-regulated protein 1	55	0.66	0.13	1.16	0.17	1.74	0.005
**O88600**	Heat shock 70 kDa protein 4	28	0.73	0.13	1.26	0.11	1.72	0.001
**P41777**	Nucleolar and coiled-body phosphoprotein 1	4	0.65	0.19	1.11	0.13	1.71	0.023
**P13635**	Ceruloplasmin	19	0.67	0.20	1.14	0.22	1.70	0.028
**P11442**	Clathrin heavy chain 1	103	0.77	0.10	1.28	0.15	1.67	0.001
**P30009**	Myristoylated alanine-rich C-kinase substrate	12	0.50	0.17	0.84	0.10	1.67	0.032
**Q8K1Q0**	Glycylpeptide N-tetradecanoyltransferase 1	5	0.87	0.15	1.45	0.34	1.66	0.016
**P62425**	60S ribosomal protein L7a	13	0.70	0.20	1.16	0.15	1.65	0.012
**P48721**	Stress-70 protein, mitochondrial	67	0.75	0.14	1.23	0.25	1.64	0.015
**P63102**	14-3-3 protein zeta/delta	26	0.63	0.11	1.03	0.17	1.63	0.006
**Q68FR6**	Elongation factor 1-gamma	21	0.73	0.08	1.19	0.20	1.63	0.002
**P20673**	Argininosuccinate lyase	42	0.69	0.14	1.13	0.27	1.62	0.035
**P04276**	Vitamin D-binding protein	15	0.78	0.14	1.24	0.33	1.60	0.031
**P63039**	60 kDa heat shock protein, mitochondrial	161	0.87	0.30	1.39	0.34	1.60	0.049
**Q9EPH8**	Polyadenylate-binding protein 1	24	0.82	0.14	1.30	0.36	1.59	0.038
**P08082**	Clathrin light chain B	3	0.67	0.13	1.07	0.10	1.59	0.005
**P11232**	Thioredoxin	7	0.69	0.12	1.10	0.30	1.58	0.032
**P19945**	60S acidic ribosomal protein P0	24	0.68	0.12	1.08	0.14	1.58	0.007
**P62634**	Cellular nucleic acid-binding protein	5	0.71	0.18	1.11	0.08	1.56	0.012
**P28064**	Proteasome subunit beta type-8	6	0.76	0.16	1.18	0.22	1.56	0.019
**P02401**	60S acidic ribosomal protein P2	31	0.71	0.09	1.11	0.16	1.56	0.004
**B5DFC8**	Eukaryotic translation initiation factor 3 subunit C	14	0.85	0.12	1.31	0.10	1.55	0.002
**Q5RKI1**	Eukaryotic initiation factor 4A-II	22	0.84	0.12	1.31	0.11	1.55	0.002
**P81795**	Eukaryotic translation initiation factor 2 subunit 3	11	0.77	0.09	1.19	0.31	1.54	0.021
**O08629**	Transcription intermediary factor 1-beta	8	0.82	0.21	1.24	0.22	1.51	0.036
**Q62667**	Major vault protein	19	0.77	0.08	1.13	0.16	1.47	0.007
**P38656**	Lupus La protein homolog	7	0.83	0.16	1.21	0.16	1.47	0.017
**Q4V7C6**	GMP synthase [glutamine-hydrolyzing]	7	0.78	0.19	1.15	0.13	1.47	0.025
**Q5XIU5**	Proteasome inhibitor PI31 subunit	2	0.85	0.20	1.25	0.18	1.46	0.027
**P62250**	40S ribosomal protein S16	13	0.83	0.17	1.22	0.18	1.46	0.022
**Q08163**	Adenylyl cyclase-associated protein 1	9	0.85	0.23	1.23	0.20	1.45	0.044
**P04256**	Heterogeneous nuclear ribonucleoprotein A1	18	0.86	0.02	1.24	0.26	1.45	0.012

**A0JPM9**	Eukaryotic translation initiation factor 3 subunit J	5	0.80	0.22	1.16	0.16	1.45	0.047
**Q6AXS5**	Plasminogen activator inhibitor 1 RNA-binding protein	12	0.72	0.16	1.05	0.19	1.45	0.036
**Q6P799**	Seryl-tRNA synthetase, cytoplasmic	14	0.84	0.15	1.21	0.26	1.45	0.045
**Q07205**	Eukaryotic translation initiation factor 5	10	0.80	0.11	1.13	0.18	1.40	0.023
**P62815**	V-type proton ATPase subunit B, brain isoform	14	0.82	0.20	1.14	0.15	1.39	0.042
**Q9EQS0**	Transaldolase	15	0.73	0.09	0.99	0.13	1.37	0.012
**P63086**	Mitogen-activated protein kinase 1	5	0.83	0.08	1.12	0.14	1.36	0.009
**P38650**	Cytoplasmic dynein 1 heavy chain 1	51	0.76	0.07	1.03	0.14	1.36	0.011
**Q63347**	26S protease regulatory subunit 7	15	0.88	0.06	1.19	0.18	1.35	0.013
**P62859**	40S ribosomal protein S28	7	0.79	0.14	1.07	0.16	1.35	0.036
**Q6P502**	T-complex protein 1 subunit gamma	34	0.92	0.14	1.24	0.09	1.34	0.013
**Q63945**	Protein SET	9	0.77	0.15	1.03	0.15	1.34	0.049
**Q4FZY0**	EF-hand domain-containing protein D2	4	0.84	0.13	1.08	0.15	1.30	0.043
**Q6P7P5**	Basic leucine zipper and W2 domain-containing protein 1	7	0.79	0.14	1.03	0.09	1.30	0.041
**P68511**	14-3-3 protein eta	15	0.85	0.10	1.09	0.12	1.29	0.020
**P12001**	60S ribosomal protein L18	9	0.83	0.13	1.07	0.09	1.29	0.025
**P38659**	Protein disulfide-isomerase A4	45	0.86	0.14	1.09	0.09	1.27	0.039
**Q505J9**	ATPase family AAA domain-containing protein 1	2	0.93	0.08	1.14	0.16	1.24	0.044
**P29314**	40S ribosomal protein S9	13	0.86	0.10	1.06	0.08	1.24	0.022
**P18395**	Cold shock domain-containing protein E1	3	0.82	0.11	1.01	0.01	1.22	0.026
**Q9ER24**	Ataxin-10	4	0.91	0.08	1.10	0.05	1.21	0.009
**Q6RUV5**	Ras-related C3 botulinum toxin substrate 1	4	0.88	0.11	1.04	0.03	1.18	0.042
**Q6AXS3**	Protein DEK	4	0.93	0.12	1.09	0.05	1.18	0.043

Supplementary Table 1C: *iTRAQ-based proteomic comparison of liver proteins in vehicle control- and APAP-treated rats (72h timepoint).* Proteins with expression that was different (raw P < 0.05) between control and APAP-treated rats at 72h are listed. Mean expression values relative to a common pool are given for n=4 animals. Proteins are ordered according to the ratio between the 72h and control groups (lowest to highest) such that proteins with expression that was most markedly reduced at 72h appear at the top of the list.

[a]Average number of peptides used for quantification across the four individual iTRAQ runs.

[b]Uncorrected raw P value.

Uniprot Accession	Name	Peptides[a]	Vehicle control		72h		72h/ctrl	P-value[b]
			Mean	SD	Mean	SD	Ratio	
**Proteins reduced at 72h**								
**P09606**	Glutamine synthetase	17	2.73	0.78	0.06	0.03	0.02	<0.001
**P08683**	Cytochrome P450 2C11	51	2.53	0.52	0.16	0.09	0.06	<0.001
**P49890**	Estrogen sulfotransferase, isoform 6	18	3.22	0.91	0.22	0.15	0.07	<0.001
**P14141**	Carbonic anhydrase 3	70	1.99	1.30	0.18	0.20	0.09	0.007
**P36365**	Dimethylaniline monooxygenase [N-oxide-forming] 1	13	2.34	0.94	0.22	0.15	0.10	0.002
**P02761**	Major urinary protein	22	2.27	0.42	0.30	0.30	0.13	0.006
**Q9ES38**	Bile acyl-CoA synthetase	26	2.80	0.86	0.39	0.39	0.14	0.007
**P17988**	Sulfotransferase 1A1	30	1.99	0.34	0.28	0.16	0.14	0.001
**P00502**	Glutathione S-transferase alpha-1	51	1.57	1.21	0.28	0.27	0.18	0.015
**Q03336**	Regucalcin	45	1.98	0.18	0.36	0.38	0.18	0.011
**P05183**	Cytochrome P450 3A2	15	2.49	0.97	0.46	0.24	0.18	0.009
**P16303**	Carboxylesterase 3	33	1.85	0.85	0.36	0.33	0.20	0.012
**Q64565**	Alanine--glyoxylate aminotransferase 2, mitochondrial	28	1.26	0.46	0.25	0.14	0.20	0.002
**P04182**	Ornithine aminotransferase, mitochondrial	8	1.44	0.74	0.29	0.13	0.20	0.007
**P50237**	Sulfotransferase 1C1	27	1.95	0.15	0.39	0.17	0.20	<0.001
**Q64611**	Cysteine sulfinic acid decarboxylase	25	1.81	0.70	0.41	0.61	0.23	0.025
**Q07523**	Hydroxyacid oxidase 2	8	1.99	0.34	0.47	0.07	0.24	<0.001
**P12928**	Pyruvate kinase isozymes R/L	61	1.64	0.53	0.40	0.17	0.24	0.002
**P14173**	Aromatic-L-amino-acid decarboxylase	13	1.74	0.94	0.42	0.39	0.24	0.019
**P10867**	L-gulonolactone oxidase	18	2.00	0.36	0.49	0.28	0.24	0.002
**P10860**	Glutamate dehydrogenase 1, mitochondrial	118	1.63	0.34	0.40	0.35	0.25	0.019
**Q64638**	UDP-glucuronosyltransferase 1-5	25	3.79	1.58	0.97	0.93	0.26	0.022
**P50169**	Retinol dehydrogenase 3	30	1.54	0.61	0.40	0.27	0.26	0.008

**P09811**	Glycogen phosphorylase, liver form	76	1.40	0.26	0.38	0.15	0.27	0.002
**P25093**	Fumarylacetoacetase	48	1.45	0.12	0.41	0.29	0.29	0.012
**Q5U2Q3**	Ester hydrolase C11orf54 homolog	12	2.00	0.36	0.58	0.29	0.29	0.011
**P02692**	Fatty acid-binding protein, liver	69	1.94	0.49	0.57	0.18	0.29	0.002
**Q4KLP0**	Probable 2-oxoglutarate dehydrogenase E1 component DHKTD1, mitochondrial	35	1.59	0.56	0.48	0.26	0.30	0.015
**P55051**	Fatty acid-binding protein, brain	9	1.61	0.57	0.49	0.40	0.30	0.024
**Q4KLZ6**	Bifunctional ATP-dependent dihydroxyacetone kinase/FAD-AMP lyase (cyclizing)	64	1.70	0.39	0.52	0.25	0.31	0.013
**Q497B0**	Omega-amidase NIT2	34	1.58	0.09	0.49	0.24	0.31	0.003
**P23680**	Serum amyloid P-component	4	1.61	0.24	0.50	0.34	0.31	0.014
**P55159**	Serum paraoxonase/arylesterase 1	17	1.68	0.55	0.53	0.36	0.32	0.025
**P07872**	Peroxisomal acyl-coenzyme A oxidase 1	42	1.33	0.25	0.43	0.28	0.32	0.017
**P23457**	3-alpha-hydroxysteroid dehydrogenase	48	1.51	0.36	0.49	0.41	0.32	0.035
**P30839**	Fatty aldehyde dehydrogenase	36	1.74	0.55	0.56	0.37	0.32	0.018
**Q9QYU4**	Thiomorpholine-carboxylate dehydrogenase	9	1.31	0.25	0.44	0.24	0.33	0.010
**P16638**	ATP-citrate synthase	65	1.93	0.71	0.65	0.21	0.34	0.006
**P20070**	NADH-cytochrome b5 reductase 3	26	1.62	0.42	0.57	0.21	0.35	0.005
**Q920P0**	L-xylulose reductase	12	1.44	0.45	0.51	0.18	0.35	0.004
**Q07071**	Glucokinase regulatory protein	27	1.71	0.64	0.60	0.35	0.35	0.029
**P29147**	D-beta-hydroxybutyrate dehydrogenase, mitochondrial	42	1.40	0.23	0.50	0.47	0.36	0.047
**Q8CHM7**	2-hydroxyacyl-CoA lyase 1	28	1.66	0.35	0.61	0.40	0.36	0.021
**P11497**	Acetyl-CoA carboxylase 1	44	1.46	0.57	0.53	0.18	0.37	0.010
**P57113**	Maleylacetoacetate isomerase	36	1.58	0.34	0.58	0.42	0.37	0.033
**B0BNE5**	S-formylglutathione hydrolase	23	1.32	0.33	0.49	0.16	0.37	0.003
**Q6DGG1**	Alpha/beta hydrolase domain-containing protein 14B	12	1.36	0.25	0.51	0.29	0.37	0.042
**P12785**	Fatty acid synthase	155	1.79	1.03	0.67	0.11	0.38	0.030
**P12938**	Cytochrome P450 2D3	46	1.52	0.33	0.57	0.11	0.38	0.001
**Q9R063**	Peroxiredoxin-5, mitochondrial	21	1.83	0.94	0.70	0.23	0.38	0.032
**A0JPQ8**	Alkylglycerol monooxygenase	6	1.64	0.20	0.63	0.19	0.38	0.001
**P70712**	Kynureninase	19	1.41	0.32	0.54	0.19	0.38	0.007

**P70473**	Alpha-methylacyl-CoA racemase	18	1.07	0.04	0.41	0.30	0.39	0.028
**Q68FU3**	Electron transfer flavoprotein subunit beta	29	1.17	0.22	0.46	0.24	0.39	0.010
**P19112**	Fructose-1,6-bisphosphatase 1	72	1.71	0.38	0.67	0.38	0.39	0.018
**Q66HG4**	Aldose 1-epimerase	5	1.37	0.18	0.54	0.12	0.39	<0.001
**P24329**	Thiosulfate sulfurtransferase	41	1.33	0.20	0.52	0.41	0.39	0.042
**Q02253**	Methylmalonate-semialdehyde dehydrogenase [acylating], mitochondrial	124	1.22	0.24	0.48	0.39	0.39	0.044
**P00884**	Fructose-bisphosphate aldolase B	129	1.31	0.33	0.52	0.39	0.40	0.047
**P29411**	GTP:AMP phosphotransferase, mitochondrial	7	1.41	0.24	0.56	0.11	0.40	0.001
**O35077**	Glycerol-3-phosphate dehydrogenase [NAD+], cytoplasmic	39	1.45	0.42	0.58	0.32	0.40	0.026
**P97612**	Fatty-acid amide hydrolase 1	26	1.67	0.31	0.67	0.50	0.40	0.037
**P52873**	Pyruvate carboxylase, mitochondrial	134	1.16	0.13	0.47	0.35	0.40	0.042
**P22791**	Hydroxymethylglutaryl-CoA synthase, mitochondrial	106	1.46	0.40	0.60	0.42	0.41	0.050
**P05544**	Serine protease inhibitor A3L	30	1.22	0.12	0.50	0.43	0.41	0.042
**Q02974**	Ketohexokinase	25	1.56	0.28	0.64	0.31	0.41	0.010
**P18163**	Long-chain-fatty-acid--CoA ligase 1	78	1.54	0.13	0.65	0.48	0.42	0.044
**P22734**	Catechol O-methyltransferase	46	1.49	0.39	0.63	0.37	0.42	0.034
**Q62730**	Estradiol 17-beta-dehydrogenase 2	17	1.72	0.47	0.74	0.42	0.43	0.029
**P06214**	Delta-aminolevulinic acid dehydratase	35	1.50	0.26	0.65	0.13	0.43	0.001
**Q68FP2**	Serum paraoxonase/lactonase 3	16	1.43	0.10	0.62	0.19	0.43	0.003
**P08503**	Medium-chain specific acyl-CoA dehydrogenase, mitochondrial	26	1.49	0.32	0.65	0.32	0.44	0.015
**P05545**	Serine protease inhibitor A3K	34	1.50	0.50	0.66	0.38	0.44	0.035
**P13803**	Electron transfer flavoprotein subunit alpha, mitochondrial	50	1.29	0.23	0.57	0.37	0.44	0.045
**P30713**	Glutathione S-transferase theta-2	19	1.34	0.31	0.60	0.26	0.45	0.020
**P46720**	Solute carrier organic anion transporter family member 1A1	4	1.41	0.30	0.63	0.23	0.45	0.008
**P46953**	3-hydroxyanthranilate 3,4-dioxygenase	25	1.42	0.40	0.64	0.26	0.45	0.030
**P08009**	Glutathione S-transferase Yb-3	53	1.10	0.29	0.50	0.26	0.45	0.030

**Q64232**	Very-long-chain enoyl-CoA reductase	35	1.76	0.41	0.80	0.41	0.45	0.045
**O88813**	Long-chain-fatty-acid--CoA ligase 5	28	1.89	0.67	0.87	0.36	0.46	0.029
**P10760**	Adenosylhomocysteinase	54	1.29	0.20	0.61	0.24	0.47	0.010
**P41034**	Alpha-tocopherol transfer protein	16	1.28	0.15	0.61	0.19	0.47	0.004
**Q68G31**	Phenazine biosynthesis-like domain-containing protein	16	1.23	0.55	0.59	0.13	0.48	0.023
**P07953**	6-phosphofructo-2-kinase/fructose-2,6-bisphosphatase 1	13	1.47	0.11	0.71	0.17	0.48	0.001
**Q6AYT0**	Quinone oxidoreductase	9	1.37	0.36	0.67	0.24	0.49	0.023
**Q561R9**	Beta-lactamase-like protein 2	11	1.20	0.24	0.59	0.18	0.49	0.008
**P57093**	Phytanoyl-CoA dioxygenase, peroxisomal	10	1.36	0.22	0.67	0.18	0.50	0.004
**Q9Z339**	Glutathione S-transferase omega-1	10	1.42	0.40	0.70	0.22	0.50	0.026
**P62959**	Histidine triad nucleotide-binding protein 1	8	1.59	0.36	0.80	0.30	0.50	0.031
**P11348**	Dihydropteridine reductase	27	1.29	0.44	0.65	0.25	0.51	0.044
**Q99MS0**	SEC14-like protein 2	16	1.32	0.14	0.67	0.11	0.51	0.001
**Q63060**	Glycerol kinase	20	1.68	0.28	0.86	0.16	0.51	0.002
**P85971**	6-phosphogluconolactonase	12	1.56	0.23	0.80	0.10	0.51	<0.001
**Q63448**	Peroxisomal acyl-coenzyme A oxidase 3	27	1.27	0.23	0.65	0.26	0.51	0.027
**Q68FS4**	Cytosol aminopeptidase	53	1.52	0.14	0.78	0.30	0.51	0.031
**P16970**	ATP-binding cassette sub-family D member 3	13	1.69	0.46	0.87	0.35	0.52	0.032
**P45380**	Sulfate anion transporter 1	3	1.09	0.35	0.57	0.21	0.52	0.029
**Q9Z0V5**	Peroxiredoxin-4	16	1.03	0.11	0.55	0.19	0.53	0.016
**P52847**	Sulfotransferase family cytosolic 1B member 1	16	1.97	0.42	1.04	0.26	0.53	0.007
**Q66H45**	Tetratricopeptide repeat protein 36	5	1.20	0.34	0.65	0.13	0.54	0.014
**P43278**	Histone H1.0	4	1.51	0.31	0.82	0.26	0.54	0.021
**Q8CG45**	Aflatoxin B1 aldehyde reductase member 2	22	1.11	0.05	0.60	0.27	0.54	0.042
**Q6AXX6**	Redox-regulatory protein PAMM	8	1.54	0.33	0.84	0.13	0.55	0.005
**P30904**	Macrophage migration inhibitory factor	22	1.31	0.21	0.73	0.30	0.55	0.032
**P10868**	Guanidinoacetate N-methyltransferase	16	1.58	0.51	0.88	0.23	0.56	0.030
**P29117**	Peptidyl-prolyl cis-trans isomerase F, mitochondrial	8	1.25	0.26	0.70	0.29	0.56	0.044
**Q03626**	Murinoglobulin-1	65	1.26	0.20	0.70	0.22	0.56	0.014
**P51650**	Succinate-semialdehyde dehydrogenase, mitochondrial	10	1.21	0.32	0.68	0.05	0.56	0.007
**P14604**	Enoyl-CoA hydratase, mitochondrial	35	1.08	0.04	0.62	0.23	0.57	0.030

**Q6AYT9**	Acyl-coenzyme A synthetase ACSM5, mitochondrial	14	1.53	0.30	0.88	0.16	0.58	0.007
**P31044**	Phosphatidylethanolamine-binding protein 1	22	1.26	0.27	0.74	0.22	0.58	0.034
**Q8VID1**	Dehydrogenase/reductase SDR family member 4	6	1.10	0.23	0.65	0.15	0.59	0.011
**O89000**	Dihydropyrimidine dehydrogenase [NADP+]	12	1.36	0.32	0.80	0.21	0.59	0.037
**Q63150**	Dihydropyrimidinase	29	1.41	0.17	0.83	0.22	0.59	0.008
**P97852**	Peroxisomal multifunctional enzyme type 2	47	1.20	0.09	0.71	0.30	0.59	0.040
**P29266**	3-hydroxyisobutyrate dehydrogenase, mitochondrial	23	1.21	0.17	0.71	0.12	0.59	0.003
**Q5BK17**	Iodotyrosine dehalogenase 1	4	1.75	0.28	1.04	0.24	0.59	0.013
**P35738**	2-oxoisovalerate dehydrogenase subunit beta, mitochondrial	13	1.23	0.12	0.73	0.24	0.60	0.020
**Q6I7R3**	Isochorismatase domain-containing protein 1	10	1.44	0.23	0.86	0.15	0.60	0.007
**P27364**	3 beta-hydroxysteroid dehydrogenase type 5	19	1.41	0.31	0.84	0.29	0.60	0.037
**Q7TP48**	Adipocyte plasma membrane-associated protein	10	1.55	0.48	0.94	0.13	0.61	0.035
**O35952**	Hydroxyacylglutathione hydrolase, mitochondrial	14	1.36	0.35	0.83	0.06	0.61	0.009
**Q64380**	Sarcosine dehydrogenase, mitochondrial	41	1.28	0.19	0.81	0.24	0.63	0.037
**Q6AYQ8**	Acylpyruvase FAHD1, mitochondrial	10	1.15	0.22	0.74	0.15	0.64	0.023
**P46462**	Transitional endoplasmic reticulum ATPase	71	1.14	0.18	0.73	0.23	0.64	0.041
**P07633**	Propionyl-CoA carboxylase beta chain, mitochondrial	23	1.21	0.18	0.79	0.21	0.65	0.037
**Q9ES21**	Phosphatidylinositide phosphatase SAC1	9	1.33	0.25	0.90	0.16	0.67	0.030
**Q923M1**	Mitochondrial peptide methionine sulfoxide reductase	3	1.19	0.24	0.81	0.15	0.68	0.027
**Q505J8**	Phenylalanine--tRNA ligase alpha subunit	31	0.99	0.10	0.67	0.12	0.68	0.007
**B0BNG0**	Tetratricopeptide repeat protein 35	11	1.31	0.30	0.89	0.14	0.68	0.028
**P70552**	GTP cyclohydrolase 1 feedback regulatory protein	5	1.09	0.21	0.75	0.09	0.69	0.020
**P16617**	Phosphoglycerate kinase 1	54	1.09	0.24	0.76	0.17	0.69	0.046
**O88767**	Protein DJ-1	16	1.21	0.20	0.84	0.12	0.69	0.021
**P27605**	Hypoxanthine-guanine phosphoribosyltransferase	12	1.35	0.20	0.96	0.15	0.72	0.025
**Q64591**	2,4-dienoyl-CoA reductase, mitochondrial	23	1.02	0.10	0.75	0.14	0.73	0.023
**O35331**	Pyridoxal kinase	13	1.22	0.21	0.91	0.13	0.75	0.045
**P35433**	Amidophosphoribosyltransferase	2	1.25	0.17	0.94	0.06	0.75	0.008

**Q9R1Z0**	Voltage-dependent anion-selective channel protein 3	8	1.17	0.16	0.90	0.11	0.77	0.027
**P17625**	Glycogen [starch] synthase, liver	5	1.10	0.11	0.85	0.16	0.77	0.048
**B2RYT9**	Translational activator of cytochrome c oxidase 1	3	1.10	0.10	0.88	0.13	0.80	0.042
**Q641Y0**	Dolichyl-diphosphooligosaccharide--protein glycosyltransferase 48 kDa subunit	16	1.18	0.12	0.97	0.07	0.82	0.021
**Q6AY78**	Solute carrier family 22 member 18	4	1.00	0.11	0.83	0.05	0.82	0.025
**Q4G064**	2-methoxy-6-polyprenyl-1,4-benzoquinol methylase, mitochondrial	6	1.07	0.10	0.89	0.07	0.83	0.027
**Q8VI04**	Isoaspartyl peptidase/L-asparaginase	7	0.99	0.07	0.83	0.04	0.84	0.006
**Q9Z1N4**	3'(2'),5'-bisphosphate nucleotidase 1	4	0.94	0.07	0.83	0.05	0.88	0.044
**Proteins increased at 72h**								
**P01048**	T-kininogen 1	15	0.25	0.21	1.91	0.92	7.52	0.009
**P02764**	Alpha-1-acid glycoprotein	6	0.31	0.37	1.91	0.33	6.13	0.017
**P42930**	Heat shock protein beta-1	10	0.12	0.11	0.67	0.33	5.46	0.006
**P11980**	Pyruvate kinase isozymes M1/M2	19	0.44	0.09	2.32	1.65	5.28	0.004
**P31000**	Vimentin	32	0.51	0.15	2.40	1.34	4.74	0.004
**P04961**	Proliferating cell nuclear antigen	4	0.41	0.07	1.88	1.39	4.62	0.005
**P06866**	Haptoglobin	21	0.33	0.15	1.51	0.53	4.57	0.002
**P38918**	Aflatoxin B1 aldehyde reductase member 3	42	0.20	0.17	0.87	0.66	4.29	0.042
**Q07439**	Heat shock 70 kDa protein 1A/1B	56	0.21	0.20	0.81	0.35	3.93	0.020
**P30009**	Myristoylated alanine-rich C-kinase substrate	12	0.50	0.17	1.90	1.07	3.76	0.031
**P69897**	Tubulin beta-5 chain	75	0.53	0.17	1.83	0.76	3.48	0.004
**P11762**	Galectin-1	4	0.73	0.12	2.38	0.75	3.29	0.001
**P20059**	Hemopexin	24	0.45	0.12	1.47	0.25	3.26	<0.001
**P04906**	Glutathione S-transferase P	12	0.26	0.22	0.84	0.56	3.21	0.034
**P05370**	Glucose-6-phosphate 1-dehydrogenase	15	0.38	0.22	1.16	0.21	3.01	0.011
**P07150**	Annexin A1	12	0.55	0.23	1.62	0.67	2.96	0.015
**Q62812**	Myosin-9	161	0.57	0.26	1.69	0.72	2.96	0.017
**Q5XFX0**	Transgelin-2	13	0.55	0.23	1.61	0.49	2.95	0.010
**Q3B8Q1**	Nucleolar RNA helicase 2	7	0.48	0.20	1.40	0.38	2.90	0.004
**P07335**	Creatine kinase B-type	11	0.64	0.11	1.83	0.58	2.86	0.001
**P13383**	Nucleolin	21	0.47	0.15	1.30	0.42	2.80	0.004
**P17475**	Alpha-1-antiproteinase	39	0.47	0.18	1.30	0.36	2.74	0.005
**P06302**	Prothymosin alpha	6	0.70	0.47	1.88	0.95	2.70	0.038
**B0BNA5**	Coactosin-like protein	3	0.60	0.19	1.63	0.51	2.70	0.007
**Q07936**	Annexin A2	15	0.61	0.20	1.62	0.92	2.68	0.032

**P10960**	Sulfated glycoprotein 1	14	0.60	0.11	1.62	0.42	2.68	0.001
**P45592**	Cofilin-1	14	0.65	0.06	1.73	0.41	2.65	<0.001
**O35763**	Moesin	19	0.58	0.12	1.51	0.45	2.61	0.002
**P18437**	Non-histone chromosomal protein HMG-17	3	0.51	0.22	1.31	0.52	2.58	0.022
**O35821**	Myb-binding protein 1A	15	0.53	0.17	1.36	0.37	2.56	0.005
**O70199**	UDP-glucose 6-dehydrogenase	38	0.32	0.10	0.80	0.29	2.53	0.011
**P14480**	Fibrinogen beta chain	25	0.52	0.24	1.24	0.41	2.40	0.033
**Q9Z2G8**	Nucleosome assembly protein 1-like 1	5	0.72	0.14	1.72	0.25	2.39	0.001
**P16391**	RT1 class I histocompatibility antigen, AA alpha chain	6	0.60	0.37	1.43	0.26	2.38	0.024
**P13084**	Nucleophosmin	14	0.51	0.21	1.22	0.39	2.38	0.026
**P13221**	Aspartate aminotransferase, cytoplasmic	30	0.51	0.20	1.12	0.28	2.22	0.013
**Q6URK4**	Heterogeneous nuclear ribonucleoprotein A3	20	0.56	0.07	1.20	0.05	2.14	<0.001
**P63102**	14-3-3 protein zeta/delta	26	0.63	0.11	1.34	0.35	2.13	0.004
**P52555**	Endoplasmic reticulum resident protein 29	12	0.95	0.19	1.99	0.93	2.09	0.023
**Q62667**	Major vault protein	19	0.77	0.08	1.59	0.61	2.06	0.010
**P13635**	Ceruloplasmin	19	0.67	0.20	1.36	0.23	2.03	0.008
**P82995**	Heat shock protein HSP 90-alpha	74	0.47	0.29	0.95	0.23	2.02	0.044
**P05197**	Elongation factor 2	70	0.71	0.15	1.42	0.46	2.00	0.012
**O35814**	Stress-induced-phosphoprotein 1	24	0.72	0.16	1.44	0.63	2.00	0.025
**P68255**	14-3-3 protein theta	20	0.87	0.30	1.74	0.69	2.00	0.040
**Q63525**	Nuclear migration protein nudC	9	0.51	0.16	1.02	0.36	1.99	0.028
**P24268**	Cathepsin D	12	0.86	0.30	1.71	0.28	1.97	0.006
**P34064**	Proteasome subunit alpha type-5	15	0.63	0.31	1.22	0.30	1.93	0.042
**P50503**	Hsc70-interacting protein	14	0.69	0.32	1.30	0.28	1.89	0.042
**Q66HD0**	Endoplasmin	95	0.71	0.27	1.32	0.34	1.86	0.036
**P29457**	Serpin H1	11	0.81	0.17	1.51	0.45	1.86	0.019
**Q68FR6**	Elongation factor 1-gamma	21	0.73	0.08	1.33	0.22	1.83	0.001
**P63018**	Heat shock cognate 71 kDa protein	106	0.60	0.12	1.10	0.21	1.82	0.006
**O54753**	17-beta-hydroxysteroid dehydrogenase type 6	12	0.85	0.33	1.53	0.31	1.80	0.024
**Q63617**	Hypoxia up-regulated protein 1	55	0.66	0.13	1.19	0.10	1.80	0.002
**P04639**	Apolipoprotein A-I	20	0.82	0.28	1.46	0.27	1.77	0.035
**P19945**	60S acidic ribosomal protein P0	24	0.68	0.12	1.18	0.36	1.73	0.026
**Q5BJY9**	Keratin, type I cytoskeletal 18	82	0.77	0.29	1.30	0.16	1.70	0.043
**Q5M9G3**	Caprin-1	4	0.88	0.24	1.46	0.40	1.65	0.035
**Q1JU68**	Eukaryotic translation initiation factor 3 subunit A	23	0.66	0.17	1.07	0.24	1.64	0.026

**P38656**	Lupus La protein homolog	7	0.83	0.16	1.32	0.25	1.60	0.018
**P62634**	Cellular nucleic acid-binding protein	5	0.71	0.18	1.14	0.24	1.60	0.024
**P62425**	60S ribosomal protein L7a	13	0.70	0.20	1.11	0.13	1.58	0.015
**P62828**	GTP-binding nuclear protein Ran	8	0.80	0.17	1.26	0.28	1.57	0.022
**P81795**	Eukaryotic translation initiation factor 2 subunit 3	11	0.77	0.09	1.21	0.12	1.57	0.001
**Q62651**	Delta(3,5)-Delta(2,4)-dienoyl-CoA isomerase, mitochondrial	17	0.62	0.20	0.97	0.15	1.56	0.035
**P62250**	40S ribosomal protein S16	13	0.83	0.17	1.29	0.18	1.56	0.011
**P11442**	Clathrin heavy chain 1	103	0.77	0.10	1.20	0.25	1.56	0.011
**P38650**	Cytoplasmic dynein 1 heavy chain 1	51	0.76	0.07	1.18	0.32	1.56	0.018
**O08557**	N(G),N(G)-dimethylarginine dimethylaminohydrolase 1	11	0.68	0.19	1.04	0.20	1.54	0.033
**P12346**	Serotransferrin	78	0.76	0.13	1.16	0.31	1.54	0.049
**P04644**	40S ribosomal protein S17	17	0.80	0.26	1.23	0.19	1.54	0.041
**Q08163**	Adenylyl cyclase-associated protein 1	9	0.85	0.23	1.31	0.35	1.54	0.049
**O08629**	Transcription intermediary factor 1-beta	8	0.82	0.21	1.27	0.11	1.54	0.016
**Q9JJ22**	Endoplasmic reticulum aminopeptidase 1	8	0.93	0.14	1.39	0.37	1.49	0.038
**Q9R0J8**	Legumain	3	0.94	0.13	1.39	0.19	1.48	0.008
**P69736**	Endothelial differentiation-related factor 1	2	0.81	0.20	1.20	0.18	1.48	0.040
**Q9JI85**	Nucleobindin-2	17	1.02	0.17	1.50	0.32	1.47	0.029
**P62853**	40S ribosomal protein S25	9	0.76	0.12	1.12	0.19	1.47	0.014
**Q63797**	Proteasome activator complex subunit 1	26	0.81	0.15	1.19	0.10	1.47	0.009
**Q9R1T1**	Barrier-to-autointegration factor	3	0.90	0.25	1.32	0.13	1.47	0.026
**Q4AEF8**	Coatomer subunit gamma-1	18	0.85	0.23	1.24	0.09	1.46	0.026
**P62752**	60S ribosomal protein L23a	8	0.78	0.12	1.14	0.17	1.46	0.014
**P23514**	Coatomer subunit beta	30	0.88	0.12	1.27	0.30	1.45	0.040
**P51583**	Multifunctional protein ADE2	8	1.12	0.06	1.60	0.34	1.43	0.034
**Q63945**	Protein SET	9	0.77	0.15	1.10	0.08	1.43	0.012
**Q8K1Q0**	Glycylpeptide N-tetradecanoyltransferase 1	5	0.87	0.15	1.24	0.19	1.42	0.023
**B5DFC8**	Eukaryotic translation initiation factor 3 subunit C	14	0.85	0.12	1.20	0.19	1.42	0.018
**Q5RJR8**	Leucine-rich repeat-containing protein 59	21	0.80	0.14	1.14	0.14	1.42	0.021
**P43244**	Matrin-3	12	0.91	0.07	1.28	0.09	1.41	0.001
**P25113**	Phosphoglycerate mutase 1	20	0.92	0.10	1.30	0.25	1.41	0.024
**P41123**	60S ribosomal protein L13	16	0.76	0.10	1.06	0.13	1.40	0.011
**Q3T1J1**	Eukaryotic translation initiation factor 5A-1	13	0.84	0.08	1.16	0.22	1.39	0.021
**P68511**	14-3-3 protein eta	15	0.85	0.10	1.18	0.25	1.39	0.036

**P21531**	60S ribosomal protein L3	23	0.80	0.13	1.11	0.20	1.39	0.037
**Q63584**	Transmembrane emp24 domain-containing protein 10	11	0.90	0.07	1.23	0.26	1.37	0.030
**P63086**	Mitogen-activated protein kinase 1	5	0.83	0.08	1.12	0.12	1.36	0.006
**Q641Z6**	EH domain-containing protein 1	20	0.87	0.03	1.17	0.06	1.35	<0.001
**P02401**	60S acidic ribosomal protein P2	31	0.71	0.09	0.95	0.06	1.34	0.008
**Q66H80**	Coatomer subunit delta	13	0.91	0.15	1.22	0.16	1.34	0.030
**P28023**	Dynactin subunit 1	6	0.85	0.11	1.12	0.14	1.31	0.020
**Q6RUV5**	Ras-related C3 botulinum toxin substrate 1	4	0.88	0.11	1.14	0.14	1.30	0.025
**Q62871**	Cytoplasmic dynein 1 intermediate chain 2	6	0.89	0.15	1.14	0.13	1.28	0.048
**Q5M7W5**	Microtubule-associated protein 4	8	0.89	0.09	1.14	0.13	1.27	0.020
**P85125**	Polymerase I and transcript release factor	5	0.85	0.03	1.07	0.05	1.26	<0.001
**P85972**	Vinculin	35	0.85	0.10	1.07	0.15	1.26	0.047
**Q8CFN2**	Cell division control protein 42 homolog	8	0.93	0.10	1.14	0.08	1.23	0.014
**P29314**	40S ribosomal protein S9	13	0.86	0.10	1.03	0.09	1.20	0.048
**Q5U211**	Sorting nexin-3	5	0.93	0.08	1.07	0.08	1.16	0.040
**Q62991**	Sec1 family domain-containing protein 1	8	0.98	0.05	1.14	0.04	1.16	0.002
**O35142**	Coatomer subunit beta'	9	0.93	0.05	1.04	0.04	1.11	0.020

Supplementary Table 1D: *iTRAQ-based proteomic comparison of liver proteins in vehicle control- and APAP-treated rats (96h timepoint).* Proteins with expression that was different (raw P < 0.05) between control and APAP-treated rats at 96h are listed. Mean expression values relative to a common pool are given for n=3 animals. Proteins are ordered according to the ratio between the 96h and control groups (lowest to highest) such that proteins with expression that was most markedly reduced at 96h appear at the top of the list.

[a]Average number of peptides used for quantification across the four individual iTRAQ runs.

[b]Uncorrected raw P value.

Uniprot Accession	Name	Peptides[a]	Vehicle control		96h		96h/ctrl	P-value[b]
			Mean	SD	Mean	SD	Ratio	
**Proteins reduced at 96h**								
**P08683**	Cytochrome P450 2C11	51	2.53	0.52	0.09	0.04	0.03	<0.001
**P49890**	Estrogen sulfotransferase, isoform 6	18	3.22	0.91	0.11	0.08	0.03	<0.001
**P09606**	Glutamine synthetase	17	2.73	0.78	0.10	0.03	0.03	<0.001
**P14141**	Carbonic anhydrase 3	70	1.99	1.30	0.10	0.08	0.05	0.004
**Q07523**	Hydroxyacid oxidase 2	8	1.99	0.34	0.12	0.08	0.06	<0.001
**P36365**	Dimethylaniline monooxygenase [N-oxide-forming] 1	13	2.34	0.94	0.24	0.05	0.10	<0.001
**P02761**	Major urinary protein	22	2.27	0.42	0.25	0.08	0.11	<0.001
**P10867**	L-gulonolactone oxidase	18	2.00	0.36	0.31	0.12	0.15	<0.001
**P50237**	Sulfotransferase 1C1	27	1.95	0.15	0.31	0.12	0.16	<0.001
**P17988**	Sulfotransferase 1A1	30	1.99	0.34	0.34	0.15	0.17	0.001
**Q9ES38**	Bile acyl-CoA synthetase	26	2.80	0.86	0.53	0.32	0.19	0.005
**P05183**	Cytochrome P450 3A2	15	2.49	0.97	0.48	0.42	0.19	0.028
**P12785**	Fatty acid synthase	155	1.79	1.03	0.36	0.07	0.20	0.009
**P04182**	Ornithine aminotransferase, mitochondrial	8	1.44	0.74	0.29	0.18	0.20	0.014
**Q62730**	Estradiol 17-beta-dehydrogenase 2	17	1.72	0.47	0.35	0.11	0.20	0.001
**Q02769**	Squalene synthase	8	1.70	0.55	0.36	0.14	0.21	0.005
**P11497**	Acetyl-CoA carboxylase 1	44	1.46	0.57	0.33	0.07	0.22	0.003
**P16638**	ATP-citrate synthase	65	1.93	0.71	0.45	0.01	0.23	0.001
**Q03336**	Regucalcin	45	1.98	0.18	0.49	0.08	0.25	<0.001
**P05369**	Farnesyl pyrophosphate synthase	27	1.50	0.39	0.39	0.35	0.26	0.025
**P22734**	Catechol O-methyltransferase	46	1.49	0.39	0.39	0.13	0.26	0.002
**P52847**	Sulfotransferase family cytosolic 1B member 1	16	1.97	0.42	0.53	0.15	0.27	0.001
**P17425**	Hydroxymethylglutaryl-CoA synthase, cytoplasmic	9	1.30	0.44	0.36	0.26	0.28	0.045
**P19225**	Cytochrome P450 2C70	19	1.56	0.65	0.45	0.35	0.29	0.041
**P12928**	Pyruvate kinase isozymes R/L	61	1.64	0.53	0.49	0.25	0.30	0.009
**Q63060**	Glycerol kinase	20	1.68	0.28	0.53	0.10	0.31	<0.001

**Q9EQ76**	Dimethylaniline monooxygenase [N-oxide-forming] 3	21	1.24	0.60	0.40	0.28	0.32	0.031
**Q68FT5**	Betaine--homocysteine S-methyltransferase 2	82	0.99	0.45	0.32	0.17	0.32	0.026
**Q5PPL3**	Sterol-4-alpha-carboxylate 3-dehydrogenase, decarboxylating	20	1.74	0.46	0.56	0.28	0.32	0.008
**P07953**	6-phosphofructo-2-kinase/fructose-2,6-bisphosphatase 1	13	1.47	0.11	0.49	0.34	0.33	0.018
**O35760**	Isopentenyl-diphosphate Delta-isomerase 1	4	1.38	0.41	0.47	0.11	0.34	0.006
**P02692**	Fatty acid-binding protein, liver	69	1.94	0.49	0.67	0.20	0.34	0.004
**P16303**	Carboxylesterase 3	33	1.85	0.85	0.64	0.38	0.35	0.050
**P18757**	Cystathionine gamma-lyase	37	1.40	0.63	0.48	0.19	0.35	0.026
**Q63276**	Bile acid-CoA:amino acid N-acyltransferase	57	1.25	0.27	0.45	0.15	0.36	0.004
**P30839**	Fatty aldehyde dehydrogenase	36	1.74	0.55	0.64	0.15	0.37	0.011
**Q64611**	Cysteine sulfinic acid decarboxylase	25	1.81	0.70	0.67	0.19	0.37	0.012
**Q5FVR2**	Thymidine phosphorylase	11	1.80	0.99	0.69	0.08	0.38	0.033
**P11030**	Acyl-CoA-binding protein	20	1.87	1.03	0.72	0.23	0.38	0.036
**P20070**	NADH-cytochrome b5 reductase 3	26	1.62	0.42	0.62	0.09	0.38	0.002
**P06214**	Delta-aminolevulinic acid dehydratase	35	1.50	0.26	0.58	0.07	0.39	<0.001
**Q5I0J9**	Putative L-aspartate dehydrogenase	17	1.35	0.44	0.53	0.12	0.39	0.017
**P18163**	Long-chain-fatty-acid--CoA ligase 1	78	1.54	0.13	0.61	0.06	0.40	<0.001
**Q8CHM7**	2-hydroxyacyl-CoA lyase 1	28	1.66	0.35	0.67	0.01	0.40	0.001
**P08290**	Asialoglycoprotein receptor 2	4	1.04	0.35	0.42	0.21	0.40	0.035
**P57093**	Phytanoyl-CoA dioxygenase, peroxisomal	10	1.36	0.22	0.55	0.23	0.41	0.012
**P23680**	Serum amyloid P-component	4	1.61	0.24	0.68	0.05	0.43	<0.001
**Q5BK17**	Iodotyrosine dehalogenase 1	4	1.75	0.28	0.75	0.42	0.43	0.017
**Q64654**	Lanosterol 14-alpha demethylase	9	1.45	0.43	0.63	0.09	0.43	0.008
**Q68FS4**	Cytosol aminopeptidase	53	1.52	0.14	0.66	0.12	0.43	<0.001
**Q07071**	Glucokinase regulatory protein	27	1.71	0.64	0.74	0.35	0.43	0.037
**P08009**	Glutathione S-transferase Yb-3	53	1.10	0.29	0.48	0.18	0.44	0.020
**P55051**	Fatty acid-binding protein, brain	9	1.61	0.57	0.71	0.31	0.44	0.041
**P85973**	Purine nucleoside phosphorylase	41	1.12	0.20	0.49	0.17	0.44	0.009
**P14669**	Annexin A3	17	1.09	0.27	0.49	0.12	0.45	0.007
**P25409**	Alanine aminotransferase 1	19	0.85	0.09	0.38	0.08	0.45	0.001

**P27364**	3 beta-hydroxysteroid dehydrogenase type 5	19	1.41	0.31	0.64	0.13	0.45	0.004
**P10860**	Glutamate dehydrogenase 1, mitochondrial	118	1.63	0.34	0.74	0.34	0.45	0.037
**P97562**	Peroxisomal acyl-coenzyme A oxidase 2	38	1.45	0.15	0.66	0.34	0.46	0.038
**O88813**	Long-chain-fatty-acid--CoA ligase 5	28	1.89	0.67	0.87	0.04	0.46	0.015
**P24329**	Thiosulfate sulfurtransferase	41	1.33	0.20	0.62	0.19	0.47	0.006
**O89000**	Dihydropyrimidine dehydrogenase [NADP+]	12	1.36	0.32	0.64	0.12	0.47	0.011
**Q920P0**	L-xylulose reductase	12	1.44	0.45	0.69	0.10	0.48	0.013
**A0JPQ8**	Alkylglycerol monooxygenase	6	1.64	0.20	0.78	0.08	0.48	<0.001
**P00884**	Fructose-bisphosphate aldolase B	129	1.31	0.33	0.64	0.10	0.49	0.006
**P13107**	Cytochrome P450 2B3	27	1.48	0.55	0.72	0.11	0.49	0.036
**Q6AXX6**	Redox-regulatory protein PAMM	8	1.54	0.33	0.75	0.24	0.49	0.015
**Q4KLZ6**	Bifunctional ATP-dependent dihydroxyacetone kinase/FAD-AMP lyase (cyclizing)	64	1.70	0.39	0.83	0.18	0.49	0.009
**Q66H45**	Tetratricopeptide repeat protein 36	5	1.20	0.34	0.59	0.21	0.49	0.036
**P12938**	Cytochrome P450 2D3	46	1.52	0.33	0.75	0.12	0.50	0.006
**P10868**	Guanidinoacetate N-methyltransferase	16	1.58	0.51	0.79	0.18	0.50	0.028
**Q5I0M2**	Nicotinate-nucleotide pyrophosphorylase [carboxylating]	11	1.28	0.42	0.64	0.13	0.50	0.035
**Q5U2Q3**	Ester hydrolase C11orf54 homolog	12	2.00	0.36	1.01	0.30	0.51	0.015
**Q63150**	Dihydropyrimidinase	29	1.41	0.17	0.72	0.33	0.51	0.021
**Q9Z339**	Glutathione S-transferase omega-1	10	1.42	0.40	0.72	0.28	0.51	0.048
**Q63448**	Peroxisomal acyl-coenzyme A oxidase 3	27	1.27	0.23	0.65	0.09	0.51	0.003
**Q9WUS0**	Adenylate kinase isoenzyme 4, mitochondrial	14	1.38	0.19	0.71	0.15	0.52	0.004
**Q9WU49**	Calcium-regulated heat stable protein 1	7	1.17	0.20	0.61	0.17	0.52	0.009
**P70712**	Kynureninase	19	1.41	0.32	0.74	0.08	0.52	0.008
**P57113**	Maleylacetoacetate isomerase	36	1.58	0.34	0.83	0.37	0.53	0.035
**Q7TP48**	Adipocyte plasma membrane-associated protein	10	1.55	0.48	0.83	0.07	0.53	0.026
**P97612**	Fatty-acid amide hydrolase 1	26	1.67	0.31	0.90	0.31	0.54	0.029
**P00173**	Cytochrome b5	31	1.37	0.20	0.76	0.14	0.55	0.005
**Q6P7R8**	Estradiol 17-beta-dehydrogenase 12	6	1.15	0.20	0.64	0.14	0.56	0.014
**P55159**	Serum paraoxonase/arylesterase 1	17	1.68	0.55	0.94	0.06	0.56	0.037

**Q64232**	Very-long-chain enoyl-CoA reductase	35	1.76	0.41	0.99	0.22	0.56	0.017
**Q02974**	Ketohexokinase	25	1.56	0.28	0.89	0.20	0.57	0.015
**Q497B0**	Omega-amidase NIT2	34	1.58	0.09	0.90	0.26	0.57	0.008
**Q9QZH8**	Arylacetamide deacetylase	6	1.64	0.55	0.93	0.07	0.57	0.038
**Q9QZX8**	Solute carrier organic anion transporter family member 1B2	3	1.36	0.40	0.78	0.05	0.57	0.021
**P04937**	Fibronectin	27	0.74	0.22	0.43	0.01	0.58	0.041
**Q6AXM8**	Serum paraoxonase/arylesterase 2	8	1.10	0.24	0.65	0.19	0.59	0.046
**P52873**	Pyruvate carboxylase, mitochondrial	134	1.16	0.13	0.68	0.14	0.59	0.005
**B0BNG0**	Tetratricopeptide repeat protein 35	11	1.31	0.30	0.77	0.15	0.59	0.022
**P29147**	D-beta-hydroxybutyrate dehydrogenase, mitochondrial	42	1.40	0.23	0.83	0.28	0.59	0.033
**O35331**	Pyridoxal kinase	13	1.22	0.21	0.73	0.20	0.60	0.032
**P41034**	Alpha-tocopherol transfer protein	16	1.28	0.15	0.77	0.15	0.60	0.008
**P25093**	Fumarylacetoacetase	48	1.45	0.12	0.88	0.24	0.61	0.015
**Q498D5**	Regulator of microtubule dynamics protein 2	8	1.34	0.14	0.83	0.34	0.62	0.044
**Q9QYU4**	Thiomorpholine-carboxylate dehydrogenase	9	1.31	0.25	0.81	0.25	0.62	0.039
**Q06647**	ATP synthase subunit O, mitochondrial	35	1.09	0.27	0.67	0.08	0.62	0.043
**P0C2X9**	Delta-1-pyrroline-5-carboxylate dehydrogenase, mitochondrial	43	1.30	0.10	0.81	0.23	0.62	0.016
**P09456**	cAMP-dependent protein kinase type I-alpha regulatory subunit	7	1.22	0.20	0.76	0.14	0.62	0.018
**Q6DGG1**	Alpha/beta hydrolase domain-containing protein 14B	12	1.36	0.25	0.85	0.20	0.63	0.031
**P08503**	Medium-chain specific acyl-CoA dehydrogenase, mitochondrial	26	1.49	0.32	0.94	0.13	0.63	0.024
**P70473**	Alpha-methylacyl-CoA racemase	18	1.07	0.04	0.68	0.19	0.64	0.024
**Q5M876**	Aspartoacylase-2	5	1.35	0.26	0.88	0.04	0.65	0.013
**Q68FP2**	Serum paraoxonase/lactonase 3	16	1.43	0.10	0.95	0.13	0.66	0.003
**Q99MS0**	SEC14-like protein 2	16	1.32	0.14	0.88	0.19	0.67	0.020
**Q66HG4**	Aldose 1-epimerase	5	1.37	0.18	0.93	0.13	0.68	0.015
**Q6I7R3**	Isochorismatase domain-containing protein 1	10	1.44	0.23	0.97	0.06	0.68	0.017
**Q63524**	Transmembrane emp24 domain-containing protein 2	8	1.29	0.21	0.89	0.09	0.69	0.018
**P41562**	Isocitrate dehydrogenase [NADP] cytoplasmic	59	1.21	0.08	0.88	0.15	0.72	0.014

**P17764**	Acetyl-CoA acetyltransferase, mitochondrial	60	1.28	0.07	0.93	0.15	0.72	0.009
**Q9Z1W6**	Protein LYRIC	7	1.17	0.14	0.87	0.05	0.75	0.011
**P27605**	Hypoxanthine-guanine phosphoribosyltransferase	12	1.35	0.20	1.01	0.06	0.75	0.035
**Q64057**	Alpha-aminoadipic semialdehyde dehydrogenase	55	1.17	0.20	0.89	0.07	0.76	0.050
**P60892**	Ribose-phosphate pyrophosphokinase 1	6	1.08	0.15	0.84	0.04	0.78	0.035
**P85108**	Tubulin beta-2A chain	72	1.11	0.05	0.88	0.12	0.79	0.021
**O88941**	Mannosyl-oligosaccharide glucosidase	12	1.13	0.13	0.89	0.10	0.79	0.044
**P11507**	Sarcoplasmic/endoplasmic reticulum calcium ATPase 2	30	1.01	0.10	0.81	0.07	0.80	0.028
**Q7TQ16**	Cytochrome b-c1 complex subunit 8	4	0.99	0.06	0.80	0.06	0.81	0.010
**P35738**	2-oxoisovalerate dehydrogenase subunit beta, mitochondrial	13	1.23	0.12	1.03	0.05	0.84	0.039
**B2RYT9**	Translational activator of cytochrome c oxidase 1	3	1.10	0.10	0.93	0.05	0.84	0.043
**Q62920**	PDZ and LIM domain protein 5	5	1.00	0.03	0.93	0.03	0.93	0.019
**Proteins increased at 96h**								
**P04906**	Glutathione S-transferase P	12	0.26	0.22	3.37	1.12	12.94	0.002
**P38918**	Aflatoxin B1 aldehyde reductase member 3	42	0.20	0.17	2.42	0.14	11.89	0.002
**P05982**	NAD(P)H dehydrogenase [quinone] 1	8	0.50	0.12	3.08	2.86	6.14	0.044
**O70199**	UDP-glucose 6-dehydrogenase	38	0.32	0.10	1.82	0.75	5.76	0.001
**P07687**	Epoxide hydrolase 1	54	0.40	0.19	2.11	0.48	5.32	0.004
**P04903**	Glutathione S-transferase alpha-2	51	0.54	0.23	2.46	0.87	4.58	0.005
**O89049**	Thioredoxin reductase 1, cytoplasmic	17	0.42	0.29	1.57	0.29	3.78	0.026
**P08430**	UDP-glucuronosyltransferase 1-6	40	0.58	0.23	2.06	0.36	3.52	0.003
**Q9Z0U5**	Aldehyde oxidase	52	0.68	0.20	2.21	0.58	3.23	0.002
**P05370**	Glucose-6-phosphate 1-dehydrogenase	15	0.38	0.22	1.20	0.33	3.13	0.028
**P13383**	Nucleolin	21	0.47	0.15	1.43	0.11	3.07	0.001
**Q62651**	Delta(3,5)-Delta(2,4)-dienoyl-CoA isomerase, mitochondrial	17	0.62	0.20	1.76	0.44	2.83	0.005
**P48508**	Glutamate--cysteine ligase regulatory subunit	9	0.68	0.26	1.74	0.31	2.57	0.021
**P23965**	Enoyl-CoA delta isomerase 1, mitochondrial	18	0.59	0.37	1.46	0.30	2.50	0.046
**P18437**	Non-histone chromosomal protein HMG-17	3	0.51	0.22	1.26	0.52	2.49	0.037

**Q9Z2Y0**	Glycine N-acyltransferase-like protein Keg1	6	0.79	0.21	1.94	0.24	2.45	0.003
**O35547**	Long-chain-fatty-acid--CoA ligase 4	9	0.78	0.11	1.88	0.58	2.40	0.003
**P51647**	Retinal dehydrogenase 1	29	0.85	0.45	2.00	0.05	2.35	0.040
**Q99PS8**	Histidine-rich glycoprotein	9	1.03	0.33	2.41	0.76	2.34	0.027
**P13084**	Nucleophosmin	14	0.51	0.21	1.17	0.03	2.29	0.036
**P17475**	Alpha-1-antiproteinase	39	0.47	0.18	1.08	0.28	2.28	0.022
**P13221**	Aspartate aminotransferase, cytoplasmic	30	0.51	0.20	1.15	0.05	2.26	0.016
**Q3B8Q1**	Nucleolar RNA helicase 2	7	0.48	0.20	1.06	0.16	2.21	0.021
**Q64633**	UDP-glucuronosyltransferase 1-7	23	0.72	0.20	1.58	0.26	2.20	0.009
**Q9EQS0**	Transaldolase	15	0.73	0.09	1.58	0.60	2.18	0.011
**O35821**	Myb-binding protein 1A	15	0.53	0.17	1.16	0.11	2.17	0.010
**P19468**	Glutamate--cysteine ligase catalytic subunit	22	0.87	0.17	1.74	0.13	2.01	0.002
**P06761**	78 kDa glucose-regulated protein	112	0.69	0.25	1.37	0.23	1.99	0.029
**Q6P6S9**	Ectonucleoside triphosphate diphosphohydrolase 5	12	0.79	0.26	1.55	0.40	1.96	0.026
**P70619**	Glutathione reductase (Fragment)	8	0.68	0.17	1.31	0.32	1.94	0.019
**Q6P747**	Heterochromatin protein 1-binding protein 3	4	0.91	0.19	1.75	0.51	1.93	0.019
**P18886**	Carnitine O-palmitoyltransferase 2, mitochondrial	21	0.84	0.19	1.59	0.48	1.89	0.037
**P63102**	14-3-3 protein zeta/delta	26	0.63	0.11	1.17	0.07	1.85	0.002
**Q5M827**	Pirin	3	0.78	0.33	1.45	0.20	1.85	0.048
**P48721**	Stress-70 protein, mitochondrial	67	0.75	0.14	1.39	0.32	1.84	0.013
**P31210**	3-oxo-5-beta-steroid 4-dehydrogenase	51	1.07	0.22	1.95	0.18	1.83	0.007
**P41777**	Nucleolar and coiled-body phosphoprotein 1	4	0.65	0.19	1.18	0.21	1.82	0.044
**Q6URK4**	Heterogeneous nuclear ribonucleoprotein A3	20	0.56	0.07	1.02	0.25	1.82	0.009
**P63039**	60 kDa heat shock protein, mitochondrial	161	0.87	0.30	1.58	0.22	1.82	0.027
**P97584**	Prostaglandin reductase 1	16	0.88	0.10	1.58	0.42	1.80	0.015
**Q68FR9**	Elongation factor 1-delta	19	0.75	0.20	1.35	0.43	1.79	0.040
**P11442**	Clathrin heavy chain 1	103	0.77	0.10	1.36	0.53	1.78	0.037
**P61980**	Heterogeneous nuclear ribonucleoprotein K	27	0.78	0.26	1.38	0.31	1.77	0.038
**P04785**	Protein disulfide-isomerase	89	0.89	0.30	1.57	0.32	1.76	0.046
**P70580**	Membrane-associated progesterone receptor component 1	18	0.87	0.20	1.51	0.25	1.74	0.015
**Q9Z2G8**	Nucleosome assembly protein 1-like 1	5	0.72	0.14	1.25	0.15	1.73	0.011
**P24368**	Peptidyl-prolyl cis-trans isomerase B	14	0.74	0.20	1.27	0.30	1.72	0.045

**O35814**	Stress-induced-phosphoprotein 1	24	0.72	0.16	1.23	0.31	1.71	0.029
**P05182**	Cytochrome P450 2E1	29	0.89	0.14	1.51	0.26	1.70	0.009
**P04961**	Proliferating cell nuclear antigen	4	0.41	0.07	0.69	0.16	1.70	0.021
**P24268**	Cathepsin D	12	0.86	0.30	1.47	0.32	1.70	0.046
**P10960**	Sulfated glycoprotein 1	14	0.60	0.11	1.02	0.19	1.68	0.013
**Q6AXS5**	Plasminogen activator inhibitor 1 RNA-binding protein	12	0.72	0.16	1.21	0.29	1.66	0.039
**P62752**	60S ribosomal protein L23a	8	0.78	0.12	1.26	0.07	1.62	0.004
**P11232**	Thioredoxin	7	0.69	0.12	1.11	0.05	1.60	0.009
**P17178**	Sterol 26-hydroxylase, mitochondrial	13	1.01	0.13	1.61	0.27	1.60	0.008
**P19945**	60S acidic ribosomal protein P0	24	0.68	0.12	1.08	0.16	1.59	0.017
**Q9ER34**	Aconitate hydratase, mitochondrial	46	0.78	0.23	1.23	0.08	1.58	0.029
**Q1JU68**	Eukaryotic translation initiation factor 3 subunit A	23	0.66	0.17	0.98	0.08	1.50	0.041
**Q641Y8**	ATP-dependent RNA helicase DDX1	12	0.73	0.13	1.09	0.15	1.50	0.025
**P08082**	Clathrin light chain B	3	0.67	0.13	1.01	0.09	1.50	0.022
**P38656**	Lupus La protein homolog	7	0.83	0.16	1.23	0.21	1.49	0.039
**P62919**	60S ribosomal protein L8	11	0.79	0.17	1.18	0.19	1.49	0.040
**Q63797**	Proteasome activator complex subunit 1	26	0.81	0.15	1.19	0.17	1.48	0.031
**P62161**	Calmodulin	20	0.78	0.14	1.15	0.13	1.48	0.024
**O88600**	Heat shock 70 kDa protein 4	28	0.73	0.13	1.07	0.05	1.47	0.011
**O35244**	Peroxiredoxin-6	32	0.87	0.20	1.27	0.11	1.46	0.045
**P07151**	Beta-2-microglobulin	3	0.81	0.10	1.17	0.17	1.45	0.012
**Q68FR6**	Elongation factor 1-gamma	21	0.73	0.08	1.05	0.24	1.44	0.044
**P48679**	Prelamin-A/C	35	0.78	0.07	1.12	0.25	1.43	0.043
**Q9EPH8**	Polyadenylate-binding protein 1	24	0.82	0.14	1.15	0.05	1.40	0.022
**Q5M875**	17-beta-hydroxysteroid dehydrogenase 13	22	1.09	0.17	1.51	0.14	1.39	0.024
**Q05096**	Myosin-Ib	94	1.03	0.04	1.42	0.01	1.38	<0.001
**P14942**	Glutathione S-transferase alpha-4	12	0.99	0.12	1.37	0.23	1.38	0.035
**P04256**	Heterogeneous nuclear ribonucleoprotein A1	18	0.86	0.02	1.15	0.20	1.34	0.019
**P25113**	Phosphoglycerate mutase 1	20	0.92	0.10	1.23	0.23	1.33	0.048
**P02401**	60S acidic ribosomal protein P2	31	0.71	0.09	0.93	0.11	1.31	0.042
**B5DFC8**	Eukaryotic translation initiation factor 3 subunit C	14	0.85	0.12	1.12	0.08	1.31	0.032
**Q9Z0V6**	Thioredoxin-dependent peroxide reductase, mitochondrial	10	0.87	0.13	1.13	0.10	1.30	0.045
**P62853**	40S ribosomal protein S25	9	0.76	0.12	0.97	0.05	1.27	0.045
**Q3T1J1**	Eukaryotic translation initiation factor 5A-1	13	0.84	0.08	1.03	0.04	1.23	0.016

**Q924S5**	Lon protease homolog, mitochondrial	27	1.01	0.10	1.23	0.10	1.22	0.033
**Q9ER24**	Ataxin-10	4	0.91	0.08	1.10	0.05	1.21	0.022
**Q4G061**	Eukaryotic translation initiation factor 3 subunit B	15	0.85	0.09	1.03	0.06	1.21	0.040
**P61314**	60S ribosomal protein L15	7	0.91	0.11	1.10	0.03	1.21	0.041
**Q63584**	Transmembrane emp24 domain-containing protein 10	11	0.90	0.07	1.08	0.03	1.20	0.014

Supplementary Table 2: Principle Component Analysis PC1 vs PC4 identifies a group of proteins which are highly expressed in rat livers after four daily doses of APAP (96 h group) and are therefore candidate proteins for further exploration of adaptation

Accession	Name
**P04906**	Glutathione S-transferase P
**P05982**	NAD(P)H dehydrogenase [quinone] 1
**P38918**	Aflatoxin B1 aldehyde reductase member 3
**P04903**	Glutathione S-transferase alpha-2
**P07687**	Epoxide hydrolase 1
**Q9Z0U5**	Aldehyde oxidase
**P05179**	Cytochrome P450 2C7
**P06757**	Alcohol dehydrogenase 1
**Q99PS8**	Histidine-rich glycoprotein
**P50137**	Transketolase
**P08430**	UDP-glucuronosyltransferase 1-6
**O70199**	UDP-glucose 6-dehydrogenase
**O35547**	Long-chain-fatty-acid--CoA ligase 4
**P48508**	Glutamate--cysteine ligase regulatory subunit
**P31210**	3-oxo-5-beta-steroid 4-dehydrogenase
**P04905**	Glutathione S-transferase Mu 1
**P05182**	Cytochrome P450 2E1
**P05545**	Serine protease inhibitor A3K
**P51647**	Retinal dehydrogenase 1
**Q9Z2Y0**	Glycine N-acyltransferase-like protein Keg1

Supplementary Table 3: Top 25 most perturbed canonical pathways, with lists of individual proteins contributing to the perturbation

LPS/IL-1 Mediated Inhibition of RXR Function	Superpathway of Melatonin Degradation	Melatonin Degradation I	Dopamine Degradation	NRF2-mediated Oxidative Stress Response	γ-linolenate Biosynthesis II (Animals)	Xenobiotic Metabolism Signaling	Serotonin Degradation	Nicotine Degradation II	Fatty Acid β-oxidation I	EIF2 Signaling	Estrogen Biosynthesis	Acute Phase Response Signaling
ACOX1	CYP1A2	CYP2E1	ALDH1A1	AKR1A1	ACSL1	MAPK1	AKR1A1	CYP2E1	ACADM	EIF3A	CYP1A2	APCS
ACOX2	CYP2C18	CYP1A2	ALDH2	AKR7A2	ACSL4	ALDH1A1	ALDH1A1	AOX1	ACSL1	EIF3B	CYP2C18	CP
ACSL1	CYP2C9	CYP2C18	ALDH3A2	AKR7A3	ACSL5	ALDH3A2	ALDH2	CYP1A2	ACSL4	EIF3C/EIF3CL	CYP2C9	FGA
ACSL4	CYP2D6	CYP2C9	ALDH4A1	AOX1	CYB5A	ALDH4A1	ALDH3A2	CYP2C18	ACSL5	EIF3J	CYP2D6	FGB
ACSL5	CYP2E1	CYP2D6	COMT	CAT	CYB5R3	ALDH5A1	ALDH4A1	CYP2C9	ECI1	EIF4A2	CYP2E1	FGG
ALDH1A1	CYP3A4	CYP3A4	MAOB	EPHX1	SLC27A2	CAT	DHRS4	CYP2D6	ECI2	EIF5	CYP3A4	FN1
ALDH3A2	CYP4F8	CYP4F8	Sult1a1	ERP29	SLC27A5	Ces1d	MAOB	CYP3A4	EHHADH	MAPK1	CYP4F8	HMOX1
ALDH4A1	CYP51A1	CYP51A1	SULT1B1	FMO1		CYP1A2	Sult1a1	CYP4F8	HADH	PABPC1	CYP51A1	HNRNPK
ALDH5A1	MAOB	Sult1a1	SULT1C3	GCLC		CYP2C9	SULT1B1	CYP51A1	HSD17B4	RPL15	HSD17B12	HP
CAT	Sult1a1	SULT1B1		GCLM		CYP3A4	SULT1C3	FMO1	SLC27A2	RPL18	HSD17B2	HRG
CPT2	SULT1B1	SULT1C3		GSR		FMO1	UGT1A1	FMO3	SLC27A5	RPL27A	HSD17B4	MAPK1
Cyp2a2	SULT1C3	UGT1A1		GSTA1		FMO3	UGT1A4	UGT1A1		RPL3		ORM1
CYP2C9	UGT1A1	UGT1A4		GSTA5		GCLC	UGT1A6	UGT1A4		RPL38		RBP1
CYP3A4	UGT1A4	UGT1A6		GSTM1		GSTA1	Ugt1a7c	UGT1A6		RPL8		SERPINA1
Cyp4a14	UGT1A6	Ugt1a7c		GSTM2		GSTA5	Ugt2b	Ugt1a7c		RPLP0		SERPINA3
FABP1	Ugt1a7c	Ugt2b		GSTO1		GSTM1		Ugt2b		RPLP2		STAT3
FMO1	Ugt2b			GSTP1		GSTM2				RPS16		TF
FMO3				GSTT2/GSTT2B		GSTO1				RPS18		
GSTA1				HMOX1		GSTP1				RPS21		
GSTA5				MAPK1		GSTT2/GSTT2B						
GSTM1				MGST1		HMOX1						
GSTM2				NQO1		HSP90AA1						
GSTO1				PPIB		HSP90AB1						
GSTP1				STIP1		HSP90B1						
GSTT2/GSTT2B				TXN		MAOB						
HMGCS1				TXNRD1		MGST1						
HMGCS2				VCP		NQO1						
MAOB						SULT1B1						
MGST1						SULT1C3						
SLC27A2						UGT1A1						
SLC27A5						UGT1A4						
SLCO1B3						UGT1A6						
SULT1B1						Ugt1a7c						
SULT1C3						Ugt2b						

Nicotine Degradation III	Bile Acid Biosynthesis, Neutral Pathway	Fatty Acid Activation	Thyroid Hormone Metabolism II (via Conjugation and/or Degradation)	Mitochondrial L-carnitine Shuttle Pathway	Aryl Hydrocarbon Receptor Signaling	Bupropion Degradation	Acetone Degradation I (to Methylglyoxal)	Glutathione-mediated Detoxification	Aldosterone Signaling in Epithelial Cells	PXR/RXR Activation	Superpathway of Cholesterol Biosynthesis
ALB	AKR1D1	ACSL1	Sult1a1	ACSL1	ALDH1A1	CYP1A2	CYP1A2	GSTA1	AHCY	ALDH1A1	ACAT1
AOX1	AMACR	ACSL4	SULT1B1	ACSL4	ALDH3A2	CYP2C18	CYP2C18	Gsta4	HSP90AA1	ALDH3A2	CYP51A1
C4A/C4B	BAAT	ACSL5	SULT1C3	ACSL5	ALDH4A1	CYP2C9	CYP2C9	GSTA5	HSP90AB1	CYP1A2	FDFT1
CYP1A2	CYP27A1	SLC27A2	UGT1A1	CPT2	ALDH5A1	CYP2D6	CYP2D6	GSTM1	HSP90B1	CYP2C9	FDPS
CYP2C18	CYP3A4	SLC27A5	UGT1A4	SLC27A2	CTSD	CYP2E1	CYP2E1	GSTM2	HSPA1A/HSPA1B	CYP3A4	HMGCS1
CYP2C9	SCP2		UGT1A6	SLC27A5	CYP1A2	CYP3A4	CYP3A4	GSTO1	HSPA5	GSTA1	HMGCS2
CYP2D6	SLC27A5		Ugt1a7c		GSTA1	CYP4F8	CYP4F8	GSTP1	HSPA8	GSTM1	IDI1
CYP2E1			Ugt2b		GSTA5	CYP51A1	CYP51A1	GSTT2/GSTT2B	HSPA9	GSTM2	NSDHL
CYP3A4					GSTM1			MGST1	HSPB1	HMGCS2	
CYP4F8					GSTM2				HSPD1	PRKAR1A	
CYP51A1					GSTO1				HSPE1	SLCO1B3	
HPX					GSTP1				HSPH1	UGT1A1	
ITIH3					GSTT2/GSTT2B				MAPK1		
UGT1A1					HSP90AA1						
UGT1A4					HSP90AB1						
UGT1A6					HSP90B1						
Ugt1a7c					HSPB1						
Ugt2b					MAPK1						
					MGST1						
					NQO1						

Supplementary Table 4: Transporters significantly changed in abundance over experimental timecourse compared to control data

Time (h)	Acc.no.	ID	fold change
48	P12336	GLUT-2	-0.43
48	P46720	OATP-1	-0.97
48	Q9QZX8	OATP-4	-1.03
72	P46720	OATP-1	-1.16
72	P45380	SAT-1	-0.93
72	P16970	PMP70	-0.96
96	P0C546	SLC25A42	-0.47

Supplementary Figure 1:

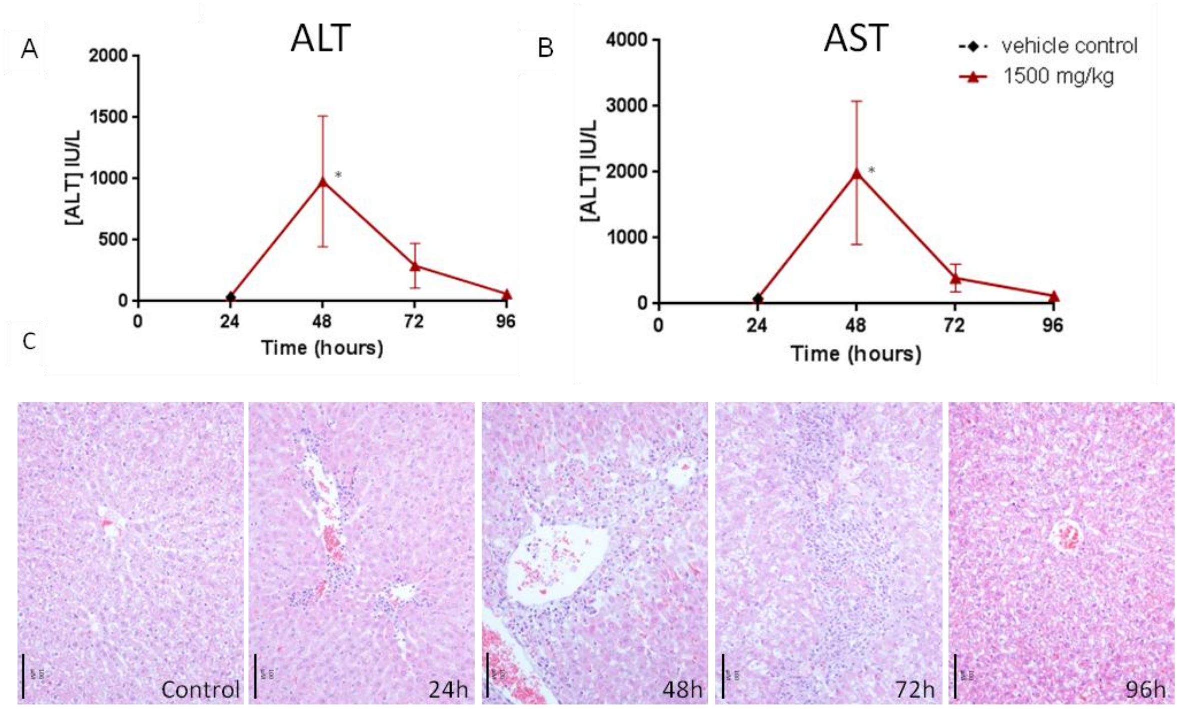

Supplementary Figure 1 Legend: Single dosed control study. Rats received a single 1500 mg/kg dose of APAP (p.o.) at 0h, with sacrifice at 24 h, 48 h, 72 h and 96 h post dose. Biomarkers of liver injury were significantly elevated at 48 h alone. Statistical analyses were performed using a one-way ANOVA with Tukey post-hoc test. * indicates P<0.05. Dose key: 1500 mg/kg, red triangles; 24h vehicle control, black diamond. A: ALT was measured in rat 24 h (43.7 IU/L, SEM 3.3), 48 h (979.6 IU/L, SEM 533.5, P=0.0458), 72 h (290.2 IU/L, SEM 184.3) and 96 h (61.6 IU/L, SEM 14.7). 24 h vehicle control values are also shown (34.2 IU/L, SEM 1.5). B: AST was measured in rat 24 h (91.5 IU/L, SEM 15.9), 48 h (1988.3 IU/L, SEM 1085.6, P=0.0378), 72 h (392.0 IU/L, SEM 211.8) and 96 h (120.8 IU/L, SEM 22.9). 24 h vehicle control values are also shown (74.8 IU/L, SEM 3.3). C: Formalin-fixed, paraffin embedded rat liver slices were stained with haematoxylin and eosin, showing progression of injury (n=4, representative images shown). Scale bar represents 100 µm.
